# Polymethoxyflavones from *Nicotiana plumbaginifolia* (Solanaceae) Exert Antinociceptive and Neuropharmacological Effects in Mice

**DOI:** 10.3389/fphar.2018.00085

**Published:** 2018-02-20

**Authors:** Md. Shafiullah Shajib, Ridwan B. Rashid, Long C. Ming, Shanta Islam, Md. Moklesur R. Sarker, Lutfun Nahar, Satyajit D. Sarker, Bidyut K. Datta, Mohammad A. Rashid

**Affiliations:** ^1^Department of Pharmacy, Stamford University Bangladesh, Dhaka, Bangladesh; ^2^Department of Pharmacy, State University of Bangladesh, Dhaka, Bangladesh; ^3^School of Pharmacy, KPJ Healthcare University College, Nilai, Malaysia; ^4^Unit for Medication Outcomes Research and Education, Pharmacy, University of Tasmania, Hobart, TAS, Australia; ^5^Faculty of Science, School of Pharmacy and Biomolecular Sciences, Liverpool John Moores University, Liverpool, United Kingdom; ^6^Department of Pharmaceutical Chemistry, Faculty of Pharmacy, University of Dhaka, Dhaka, Bangladesh

**Keywords:** *Nicotiana plumbaginifolia*, polymethoxyflavone, antinociceptive, opioid, anxiolytic, benzodiazepine

## Abstract

Polymethoxylavones (PMFs) are known to exhibit significant anti-inflammatory and neuroprotective properties. *Nicotiana plumbaginifolia*, an annual Bangladeshi herb, is rich in polymethoxyflavones that possess significant analgesic and anxiolytic activities. The present study aimed to determine the antinociceptive and neuropharmacological activities of polyoxygenated flavonoids namely- 3,3′,5,6,7,8-hexamethoxy-4′,5′-methylenedioxyflavone (**1**), 3,3′,4′,5′,5,6,7,8-octamethoxyflavone (exoticin) (**2**), 6,7,4′,5′-dimethylenedioxy-3,5,3′-trimethoxyflavone (**3**), and 3,3′,4′,5,5′,8-hexamethoxy-6,7-methylenedioxyflavone (**4**), isolated and identified from *N. plumbaginifolia*. Antinociceptive activity was assessed using the acetic-acid induced writhing, hot plate, tail immersion, formalin and carrageenan-induced paw edema tests, whereas neuropharmacological effects were evaluated in the hole cross, open field and elevated plus maze test. Oral treatment of compounds **1**, **3**, and **4** (12.5–25 mg/kg b.w.) exhibited dose-dependent and significant (*p* < 0.01) antinociceptive activity in the acetic-acid, formalin, carrageenan, and thermal (hot plate)-induced pain models. The association of ATP-sensitive K^+^ channel and opioid systems in their antinociceptive effect was obvious from the antagonist effect of glibenclamide and naloxone, respectively. These findings suggested central and peripheral antinociceptive activities of the compounds. Compound **1**, **3**, and **4** (12.5 mg/kg b.w.) demonstrated significant (*p* < 0.05) anxiolytic-like activity in the elevated plus-maze test, while the involvement of GABA_A_ receptor in the action of compound **3** and **4** was evident from the reversal effects of flumazenil. In addition, compounds **1** and **4** (12.5–25 mg/kg b.w) exhibited anxiolytic activity without altering the locomotor responses. The present study suggested that the polymethoxyflavones (**1–4**) from *N. Plumbaginifolia* could be considered as suitable candidates for the development of analgesic and anxiolytic agents.

## Introduction

Pain is an unpleasant sensory perception accompanied by physiological damage comprising actual or potential tissue injury (de Sousa, 2011). Such sensation also relies on individual's emotional state and can be exacerbated by the psychological disorders like anxiety and depression. It adversely affects the quality of life and is one of the common reasons for visiting physicians and taking medications (de Santana et al., 2015). However, conventional medicines such as non-steroidal anti-inflammatory drugs (NSAIDs), steroids, and opioid analgesics are associated with significant side effects including gastric ulcers, dependence and depression, making drug therapies more complex and difficult (Poetker and Reh, 2010; Tiwari and Singh, 2014).

Medicinal plants serve as a major source of pharmacologically active compounds that are used in the treatment of human diseases like pain and psychiatric disorders such as anxiety and depression (McCurdy and Scully, 2005; Bouayed, 2010; Saki et al., 2014). Polymethoxyflavones (PMFs) are exclusively found in citrus peels (Li et al., 2009) and some other pharmacologically significant plants (Kinoshita and Firman, 1996; Chen et al., 2011; Faqueti et al., 2016). They belong to the superfamily of flavonoids which can be classified as anthoxanthins (e.g., flavones, flavonols), flavanones, flavanonols, flavans, and anthocyanidins. PMFs are termed as flavones which contain more than one methoxy (-O-CH_3_) groups on their essential benzo-γ-pyrone skeleton, generally constituted by 15 carbons (C_6_-C_3_-C_6_), including a carbonyl group (C = O) at C-4 position (Ververidis et al., 2007; Li et al., 2008). Scientific studies revealed that PMFs might exert prominent *in vivo* or *in vitro* anti-nociceptive (Nadipelly et al., 2016), anti-inflammatory, anticarcinogenic (Li et al., 2009), cancer chemopreventive (Walle, 2007), sedative (Jin et al., 2012), anti-depressant, anxiolytic as well as benzodiazepine binding effects (Paladini et al., 1999; Abdelhalim et al., 2015) and possess excellent absorption and oral bioavailability (Li et al., 2009).

*Nicotiana plumbaginifolia* Vivane (Fam. Solanaceae) is a flowering annual herb of Bangladesh. It is used in the treatment of cuts, wounds, toothache, rheumatic swelling in the traditional system of medicines (Dangwal et al., 2010; Singh et al., 2010; Devi et al., 2014). Pharmacological studies showed that leaves of this plant possess significant analgesic and anxiolytic activities (Shahriar et al., 2015). Phytochemical analysis of the leaves of this plant afforded PMFs namely- 3,3′,5,6,7,8-hexamethoxy-4′,5′-methylenedioxyflavone (**1**), 3,3′,4′,5′,5,6,7,8-octamethoxyflavone (exoticin) (**2**), 6,7,4′,5′-dimethylenedioxy-3,5,3′-trimethoxyflavone (**3**), and 3,3′,4′,5,5′,8-hexamethoxy-6,7-methylenedioxyflavone (**4**). However, there is no report on the pharmacological actions of the PMFs isolated from *N. plumbaginifolia*. Therefore, based on the pharmacologically relevant reports of PMFs from other sources, and extractives of *N. plumbaginifolia*, PMFs from this plant were evaluated for their antinociceptive activity in the acetic acid-induced writhing, hot plate, tail immersion, formalin-induced nociception, and paw edema and carrageenan-induced paw edema test and neuropharmacological effect in open field, elevated plus maze test on mice. Experimental study along with deciphering the mechanism of actions of the experimental drugs or compounds in the living system is an important part of analytical and experimental pharmacology as well as the discovery of new therapeutic agents (Salomone, 2010). The involvement of opioid, ATP-sensitive K^+^ channel in the antinociceptive and benzodiazepine system in the anxiolytic action of the isolated PMFs were also determined in this study.

## Materials and methods

### Isolation and characterization of experimental compounds

**Figure 1:**
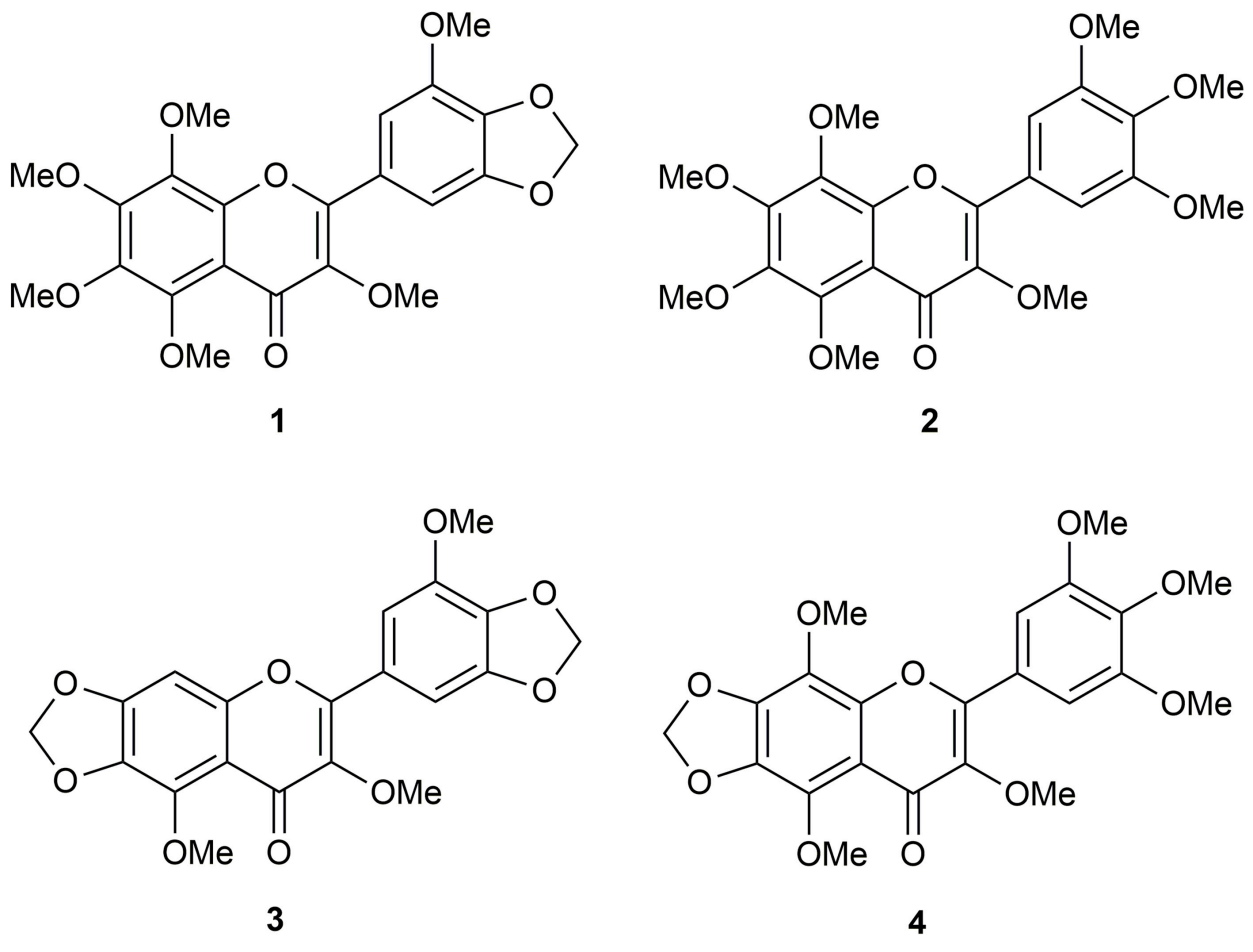
Polymethoxyflavones (PMFs) of *N. plumbaginifolia*. **1** = 3,3′,5,6,7,8-hexamethoxy-4′,5′-methylenedioxyflavone; **2** = 3,3′,4′,5′,5,6,7,8-octamethoxyflavone (exoticin); **3** = 6,7,4′,5′-dimethylenedioxy-3,5,3′-trimethoxyflavone; **4** = 3,3′,4′,5,5′,8-hexamethoxy-6,7-methylenedioxyflavone.

Repeated chromatographic separation and purification of the methanol (MeOH) extract of *N. plumbaginifolia* leaves led to the isolation and characterization of 3,3′,5,6,7,8-hexamothoxy-4′,5′-methylenedioxyflavone (**1**), 3,3′,4′,5′,5,6,7,8-octamethoxyflavone (exoticin) (**2**), 6,7,4′,5′-dimethylenedioxy-3,5,3′-trimethoxyflavone (**3**), and 3,3′,4′,5,5′,8-hexamethoxy-6,7-methylenedioxyflavone (**4**) (Figure 1), the experimental details and structural data of which are available in the literature (Shajib et al., 2017).

### Drugs and reagents

Acetic acid, potassium hydroxide (Merck Co., Darmstadt, Germany), methanol, lambda (λ)- carrageenan, formalin, pentobarbital sodium, sodium carboxymethylcellulose (Na-CMC) (Sigma, St. Louis, MO, USA), morphine sulfate (Gonoshasthaya Pharmaceuticals Ltd., Savar, Dhaka, Bangladesh), diclofenac sodium (Novartis Bangladesh Ltd., Gazipur, Dhaka, Bangladesh), naloxone hydrochloride (Hospira Australia Pty Ltd, Melbourne, Australia), glibenclamide (Square Pharmaceuticals Ltd., Gazipur, Dhaka, Bangladesh), diazepam (Square Pharmaceuticals Ltd., Gazipur, Dhaka, Bangladesh), flumazenil (Roche Bangladesh Pharmaceuticals Ltd., Dhaka, Bangladesh), physiological saline (sodium chloride 0.9% w/v) (Beximco Pharmaceuticals Ltd, Tongi, Dhaka, Bangladesh) were either purchased or obtained as gifts.

### Ethical declarations

The animals were treated according to the protocols of the Ethical Principles and Guidelines of Scientific Experiments on Animals (1995) recommended by the Swiss Academy of Medical Sciences and the Swiss Academy of Sciences. The acute oral toxicity was determined according to the recommendation (420- fixed dose procedure) of Organization for Economic Cooperation and Development (OECD). After experiments, the animals were subjected to euthanasia using pentobarbital following AVMA Guidelines for the Euthanasia of Animals of 2013 edition. All experimental methods and principles were endorsed by the Ethics Committee of Stamford University Bangladesh (SUB/IAEC/15.03). Every effort was taken to alleviate the potential sufferings of the animals.

### Animals

Pharmacological investigations were conducted on 20–25 g, 8–10 weeks old healthy male Swiss albino mice. The animals were obtained from the Animal Resources Branch of the International Center for Diarrhoeal Disease Research, Bangladesh (icddr,b). The procured animals were rehabilitated in 120 × 30 × 30 cm cages with wood shavings bedding, kept under controlled laboratory condition of 24 ± 2°C temperature, 55–60% relative humidity and 12-h light–dark cycle (light on 7:00 a.m. to 7:00 p.m.). They had free access to standard diet and water *ad libitum*. They were acclimatized in the laboratory environment for 14 days prior to the investigations. The healthcondition of the animals was checked regularly. The animals were randomly chosen and allocated into negative control, positive control, and experimental group. Each group had six animals (*n* = 6). Investigators, who were responsible for recording data, were unaware of the assigned group to avoid any experimental bias. The test animals did not have any access to food 3–4 h prior to the commencement of the experiments and experimentations were carried out between 9:00 a.m. and 5:00 p.m.

### Treatments

Animals of experimental groups were treated with single oral dose 12.5 or 25 mg/kg b.w. of the isolated compounds. The doses were selected based on pilot experiment and previously reported oral effective doses of PMFs (Jang et al., 2013). All experimental doses were formulated with physiological saline containing 0.5% Na-CMC. The control group was treated per oral (p.o) with 0.5% Na CMC as vehicle at the dose of 10 mL/kg b.w. The vehicle and experimental PMFs were administered 60 min before the experiments. PMFs have been reported to possess excellent absorption and oral bioavailability properties (Li et al., 2009), and are effective after 1 h of oral administration (Jin et al., 2012). The pharmacokinetics of drugs from oral treatment is considered to be similar to that of intraperitoneal (i.p.) treatment (Turner et al., 2011). Therefore, the oral effect of experimental PMFs has been compared with the intraperitoneally (i.e., parenteral) administered standard drugs. For the antinociceptive tests, morphine sulfate, and diclofenac sodium were used as positive control drugs at the dose of 5- and 10-mg/kg b.w., respectively. Since parenteral drugs have quicker onset of action compared to oral administration of (Turner et al., 2011) standard drugs, diclofenac (i.p.) or morphine (i.p.) were administered 30 min before the experiments. In the hot plate and tail immersion tests, naloxone was employed (i.p.) at the dose of 2 mg/kg b.w., 15 min before the administration of morphine sulfate or isolated compound to verify the possible involvement of opioid system. To evaluate the association of ATP-sensitive K^+^ channel in the analgesic effect, glibenclamide (Gbc), an ATP-sensitive K^+^ channel blocker, was administered at the dose of 10-mg/kg b.w. (i.p.), 15 min prior to administration of the isolated compound in the acetic acid-induced writhing test. Diazepam was used as the positive control in the hole cross, open field, and elevated plus maze test at 1 mg/kg (i.p.) b.w. 30 min prior to the commencement of the experiments. The participation of benzodiazepine receptor in the anxiolytic effect was justified using flumazenil, a selective benzodiazepine antagonist of GABA_A_ receptor, at the dose of 2.5 mg/kg (i.p.), 15 min before the administration of the plant isolates or diazepam in elevated plus maze test.

### Safety evaluation (acute toxicity test)

The selected doses (12.5–25 mg/kg, b.w.) of the experimental compounds were administered into the animals following the guidelines (420 fixed dose procedure) recommended by Organization for Economic Cooperation and Development (OECD) for acute toxicity study. Experimental animals were allocated into a control and eight test groups. Six animals were selected for each group (*n* = 6). Control and experimental group animals received single oral dose of vehicle (1% Na-CMC, 10 mL/kg b.w.) and test compound (12.5 or 25 mg/kg b.w.) respectively. The animals abstained from feed and water 2 h prior to treatment with vehicle or test compounds. After oral gavage, each group of mice was separately housed and had access to feed and water *ad libitum*. The animals were then observed occasionally for the next 24 h and daily for a total of 14 days. During this period, animals were observed for mortality, aberrant behavior, salivation, tremors, convulsion, and any abnormalities regarding food consumption, eyes (irritation or discharges), hair, skin (irritation, rashes, swelling or itching), and feces (physical appearance and bowel movements). At the end of the observational session, the body weight of the body weight of the animals that survived were recorded animals was recorded. Then they were sacrificed following euthanasia and vital organs of the body were isolated, examined for any abnormalities and weighed to determine any significant changes (Sehar et al., 2008; Talwar et al., 2013).

### Antinociceptive assays

#### Acetic acid-induced writhing test

The experiment acetic acid-induced pain, characterized by writhing syndrome was performed to evaluate the central and peripheral analgesic effect of the plant isolates. Experimental mice were acclimatized in the individual experimental chamber for 60 min before the test. Each mouse received intraperitoneally (i.p.) 1% w/v acetic acid (10 mL/kg) 60 min after vehicle or isolates and 30 min after diclofenac treatment. The nociceptive characteristics such as twisting of the trunk, elongation of the hind limb, stretching of the abdomen induced by the noxious stimulus were marked as writhing episodes. The time taken to begin writhing response after acetic acid administration was recorded as the onset of writhing. Beginning with the first writing response, the number of writing episodes were noted in each 10 min in the 60 min of the observational period (Bagdas et al., 2016). Analgesic activity was defined as the diminishing of writhing episodes and the percentage of inhibition of writhing was calculated as follows:

$$\begin{array}{l} \text{Inhibition}\left(\%\right)\;=\;\frac{\begin{array}{c} \text{Writhing of control group }-\\ \text{writhing of treatment group} \end{array}}{\text{Writhing of control group}}\;\times \;100 \end{array}$$

#### Hot plate test

The experiment was carried out to measure the analgesic activity of the compounds **1–4** against thermal pain threshold. Mice were gently handled and placed on the heated surface of hot plate apparatus (Eddy's hot plate, Kshitij Innovations, Haryana, India). The hot plate surface was covered by cylindrical glass and temperature was kept stable at 55 ± 1°C. The time taken to react to the thermal nociception by responses such as licking of the paw(s), jumping was recorded as latency time (Eddy and Leimbach, 1953). Animals which exhibited pre-treatment latency of 5–20 s were selected for the experiment and maximum latency time was fixed at 20 s (cut-off time) to avoid any tissue injury. A latency of each mouse was recorded just before treatment (0 min) and they served as a control of their own. Then latency was recorded at 30, 45, 60, 90, and 120 min following the administration of vehicle, isolates (p.o.) or morphine (i.p.). An increase in latency time was considered analgesic against thermal pain. The maximal possible analgesic effect was calculated as a percent (% MPE) from the following formula:

$$\begin{array}{l} \%\text{ MPE }=\;\frac{\begin{array}{c} \text{Post-treatment reaction time }-\\ \text{pre-treatment reaction time} \end{array}}{20\;-\text{ pre-treatment reaction time }}\;\times \;100 \end{array}$$

The % MPE was plotted against time and area under the curve (AUC_0–120 min_) was determined by trapezoidal rule (Bhargava et al., 1989).

#### Tail immersion test

The tail immersion test was performed to evaluate the centrally mediated analgesia of the isolated compounds **1–4** against the thermal nociceptive stimulus. Briefly, mice were immobilized gently by using “chux” and 1–2 cm of the tail of each mouse was immersed into warm water thermostatically maintained at 52 ± 1°C (Janseen et al., 1963). The rapid flick of the tail was regarded as the end-point of nociception and time taken to flick tail was noted (latency time). Mice that flicked their tail between 1.5 and 3.5 s before treatment were considered for the experiment. Then the mice were treated with vehicle, isolates or morphine and the latency was counted after 30, 45, 60, 90, and 120 min of treatment. A 20 s cut-off time was fixed in order to prevent tissue injury of mice. The pre-treatment latency of each mouse served as baseline. The maximal percent of analgesia at each observation time and (AUC_0–120 min_) was determined as described in hot plate test.

#### Formalin-induced nociception and paw edema test

The effect of isolates against chemical-induced pain was modeled by formalin test as the experiment is valid and frequently used for the evaluation of analgesic as well as anti-inflammatory agents (Hunskaar and Hole, 1987). Mice were adapted in an individual observational cage for 60 min. The experimental mice were gently held and pre-treated with vehicle, the plant isolates or morphine before formalin injection. The nociception was elicited by the treatment of 20 μL solution of 5% formalin into the right hind paw of mice. The licking and biting of the right hind paw by the mice were considered as responses of nociception and time spent for showing the responses was recorded in every 5 min for total 60 min of observation. The first 10 min of observation was defined as phase I or neurogenic phase and the next 50 min was Phase II or inflammatory phase. The vertical paw thickness of each mouse was measured before and 5 h after the treatment of formalin using a digital fine caliper (M: 091552; Shanghai Shenhan Measuring Tools Co. Ltd., Shanghai, China). Then edematogenic inflammatory response was calculated from difference between the paw thickness (Δ) of post and pre-formalin treatment (Wheeler-Aceto and Cowan, 1991; Xiao et al., 2008).

#### Carrageenan-induced paw edema test

Anti-inflammatory effect of the isolates was studied by the carrageenan-induced paw edema test. The experimental mice received vehicle, experimental compounds or diclofenac and were subjected to measure their left hind paw thickness by a Vernier caliper before carrageenan treatment. A 25 μL solution of 1% w/v lambda carrageenan was injected deliberately under the subplantar aponeurosis of the right hind paw of the mice in order to induce edema (Morris, 2003). The degree of inflammation was defined by the thickness of edema. The paw edema thickness (Δ in mm) was measured from the difference between left hind paw thickness of before and 0, 1, 2, 3, 4, 5, and 6 h after carrageenan administration. After the observing session, experimental mice were euthanized. The experimental paws were dissected and soaked overnight in 2.5 mL of physiological saline at the temperature of 0–4°C. The solution was centrifuged for 15 min at 3,000 rpm to obtain as upernatant and pellet. A volume of 0.5 mL supernatant was mixed with 2 mL solution of 0.5 M KOH (dissolved in MeOH) and incubated at 50°C for 20 min. Upon cooling, the mixture was supplemented with MeOH up to 5 mL volume and thoroughly mixed. Five minutes later the absorbance of the mixture was read at 278 nm by UV spectrophotometer (Chopade and Sayyad, 2015). The PGE2 content was represented by optical density (OD) value of the mixture.

### Neuropharmacological assays

#### Hole cross test

The effect of isolated compounds on the motor activity of the mice was evaluated by the hole cross test. The experiment was conducted following the method described by Takagi et al. (1971). Briefly, experimental mice were pre-treated with vehicle, compounds **1–4** or diazepam. They were individually placed in a box (13 × 14 × 20 cm^3^), having two compartments separated by a fixed partition. The partition contained a hole of 3 cm to facilitate the passage for mice. The mice were placed gently facing toward the hole in one of the compartments of the box. The transitions of each mouse from one compartment to another were documented for 3 min. The observation was made at 0, 30, 60, 90, and 120 min following treatment and % inhibition was determined from the transitions.

#### Open field test

Effect of the compounds on general locomotor performance was evaluated by the open field test. The procedure was carried out as described by Frye and Walf (2002). The open field apparatus (76 × 57 × 35 cm^3^) consisted a floor divided into 48 black and white colored square grids. Each square was colored alternatively. The 24 perimeter squares were considered as peripheral squares while others were central. Experimental subjects were pre-treated with compounds **1–4**, vehicle or diazepam. They were then individually placed at the center of the floor and the number of peripheral and central square crossed was documented for 5 min. The experimentation was performed in an isolated and sound-attenuated area. The total number of square crossed (ambulation) was calculated and percent of central square crossed was determined. The number of ambulation was used to determine the % inhibition of locomotion using following formula:

$$\begin{array}{l} \%\text{ Inhibition }=\;\frac{\begin{array}{c} \text{Ambulation of control group }-\\ \text{ambulation of treatment group} \end{array}}{\text{Ambulation of control group}}\;\times \;100 \end{array}$$

The increase in the number of central square crossing and percentage increase of central square crossing of mice were regarded as anxiolytic whereas reduction of such explorations was considered as anxiogenic (Prut and Belzung, 2003).

#### Elevated plus maze test

The anxiolytic action of the compounds was evaluated on elevated plus maze apparatus. The apparatus was elevated at a height of 50 cm from the floor and consisted of two elongated closed and open arms of equal dimensions (50 × 10 cm). The closed arms were enclosed by side-wall of 40 cm height. Each type of arms was arranged perpendicularly to each other. Mice were pre-treated with vehicle, isolates, or diazepam and each mouse was individually placed in the center of the maze facing their head toward one of the closed arms. The number of entries and time spent in close and open arms of the maze was recorded for the period of 5 min. The entrance of four paws of mice into an arm was regarded as an entry. The increase of the entries and percent entries, as well as exploration time in open arms of mice was considered as anxiolytic, whereas the opposite was regarded as anxiogenic (Pellow et al., 1985). The maze was cleaned with alcohol before each experimental session and the operation was carried out in a sound attenuated isolated area. The % open arm time was calculated as follows:

$$\begin{array}{l} \text{\% of open arm time }=\;\frac{\text{Time spent in open arms}}{\text{Total spent time in open and closed arms}}\times \;100 \end{array}$$

### Analysis of the possible mechanism of actions

#### Involvement of opioid receptor

To investigate the role of opioid system in the antinociceptive action of the isolated PMFs, mice were treated with a non-selective opioid antagonist, naloxone (2 mg/kg b.w., i.p.), 15 min before administration of the compound (25 mg/kg b.w., p.o.), or morphine (5 mg/kg b.w., i.p.) in the tail immersion and hot plate test. The thermal latency time of the experimental mice was documented before the treatment of standard drug or experimental compound and at 30, 45, 60, 90, 120 min of investigation period (Khan et al., 2011). The cut-off period was maintained for 20 s to avoid tissue injury as described in hot plate and tail immersion test. The documented data were assembled with the hot plate and tail immersion test result for comparison.

#### Association of the ATP-sensitive K^+^ channel pathway

The association of ATP-sensitive K^+^ channel in the pain inhibition action of the isolates in mice was evaluated as previously described (Mohamad et al., 2011; Perimal et al., 2011). Animals were pre-treated with glibenclamide (10 mg/kg b.w., i.p.), an ATP-sensitive K^+^ channel inhibitor 15 min before the administration diclofenac or effective dose (25 mg/kg b.w., p.o.) of the plant isolates. Mice were treated with 1 % w/v acetic acid (i.p.) after 60 min of the isolated compounds **1–4** administration and 30 min of diclofenac. Then, time taken to start writhing (onset time) was recorded and writhing episodes were counted in every 10 min for 60 min as described in the acetic acid-induced writhing test. These data were assembled with acid-induced writhing test result for comparison.

#### Involvement of benzodiazepine receptor

To verify the role of benzodiazepine system in the anxiolytic action of the isolated PMFs, flumazenil, a selective antagonist of GABA_A_ receptor was administered at the dose of 2.5 mg/kg (i.p.), 15 min before the administration of isolates or diazepam in elevated plus maze test (Aragão et al., 2006). After 60 min of isolated PMFs and 30 min of diazepam employment, the experimental mice were placed on the central square of maze facing to the close arm. Thenumber of entries and time spent in close and open arms was recorded for 5 min and % open arm time and entries were calculated as stated in elevated plus maze test.

### Data analysis

All results have been shown as median (*n* = 6) with range (min-max). The area under the curve (AUC) response was calculated by trapezoidal rule as an expression of the intensity of the effect. Data analysis was performed using Kruskal Wallis followed by Mann-Whitney test at the levels of significance ranging from *p* < 0.05 to 0.001 by SPSS 22 (IBM, USA).

## Results

### Acute toxicity

Oral treatment of selected doses for compounds **1–4** did not cause any abnormal effects or mortality during 14 days of observation. In addition, administration of PMFs **1–4** did not produce any injuries, sign of abnormalities and significant differences to the gross weight of the vital organs or body (Table 1) in animals.

**Table 1:** Effect of oral treatment of compounds **1–4** on gross changes of the body and vital organs of mice.

**Treatment**	**Organ weight [relative organ weight in %]**					
	**Body weight**	**Liver**	**Kidneys**	**Lungs**	**Heart**	**Spleen**
Vehicle (10 mL/kg)	22.38 (20.10–24.83)	1.53 (1.39–1.74) [6.90]	0.17 (0.14–0.19) [0.74]	0.23 (0.20–0.27) [1.03]	0.12 (0.10–0.14) [0.53]	0.14 (0.12–0.17) [0.63]
**1** (12.5 mg/kg)	22.05 (20.68–24.80)	1.56 (1.43–1.70) [6.96]	0.16 (0.13–0.18) [0.69]	0.21 (0.18–0.26) [0.95]	0.12 (0.09–0.15) [0.53]	0.15 (0.11–0.17) [0.64]
**1** (25 mg/kg	22.65 (20.73–23.54)	1.56 (1.43–1.62) [6.91]	0.16 (0.14–0.18) [0.71]	0.22 (0.19–0.25) [0.98]	0.13 (0.10–0.14) [0.55]	0.13 (0.12–0.16) [0.61]
**2** (12.5 mg/kg)	23.18 (22.12–24.55)	1.60 (1.54–1.70) [6.87]	0.17 (0.14–0.18) [0.70]	0.26 (0.22–0.28) [1.09]	0.13 (0.12–0.14) [0.55]	0.16 (0.13–0.16) [0.64]
**2** (25 mg/kg)	21.09 (20.02–24.31)	1.45 (1.36–1.75) [6.92]	0.15 (0.13–0.17) [0.68]	0.21 (0.18–0.26) [0.97]	0.13(0.11–0.15) [0.58]	0.14 (0.11–0.16) [0.62]
**3** (12.5 mg/kg)	23.31 (20.11–23.88)	1.59 (1.50–1.66) [7.01]	0.16 (0.13–0.18) [0.70]	0.26 (0.20–0.27) [1.08]	0.13 (0.10–0.14) [0.54]	0.15 (0.11–0.18) [0.65]
**3** (25 mg/kg	21.16 (20.61–22.48)	1.48 (1.45–1.50) [6.95]	0.15 (0.12–0.16) [0.69]	0.22 (0.21–0.24) [1.04]	0.13 (0.11–0.14) [0.60]	0.14 (0.12–0.16) [0.65]
**4** (12.5 mg/kg)	24.24 (23.41–24.86)	1.69 (1.62–1.75) [7.00]	0.19 (0.16–0.21) [0.77]	0.26 (0.23–0.28) [1.06]	0.14 (0.12–0.16) [0.57]	0.17 (0.14–0.18) [0.68]
**4** (25 mg/kg	21.95 (20.32–24.36)	1.52 (1.43–1.71) [6.93]	0.18 (0.16–0.22) [0.81]	0.22 (0.19–0.25) [0.98]	0.13 (0.11–0.15) [0.56]	0.15 (0.13–0.17) [0.65]

*Values are presented as median (n = 6) with range (min-max). **1** = 3,3′,5,6,7,8-hexamethoxy-4′,5′-methylenedioxyflavone; **2** = exoticin; **3** = 6,7,4′,5′-dimethylenedioxy-3,5,3′-trimethoxyflavone; **4** = 3,3′,4′,5,5′,8-hexamethoxy-6,7-methylenedioxyflavone. Relative organ weight (%) = [(organ weight/ body weight)] × 100. Values were not significant (p is not ≤ 0.05) when compared to control group*.

### Acetic acid-induced writhing

**Figure 2:**
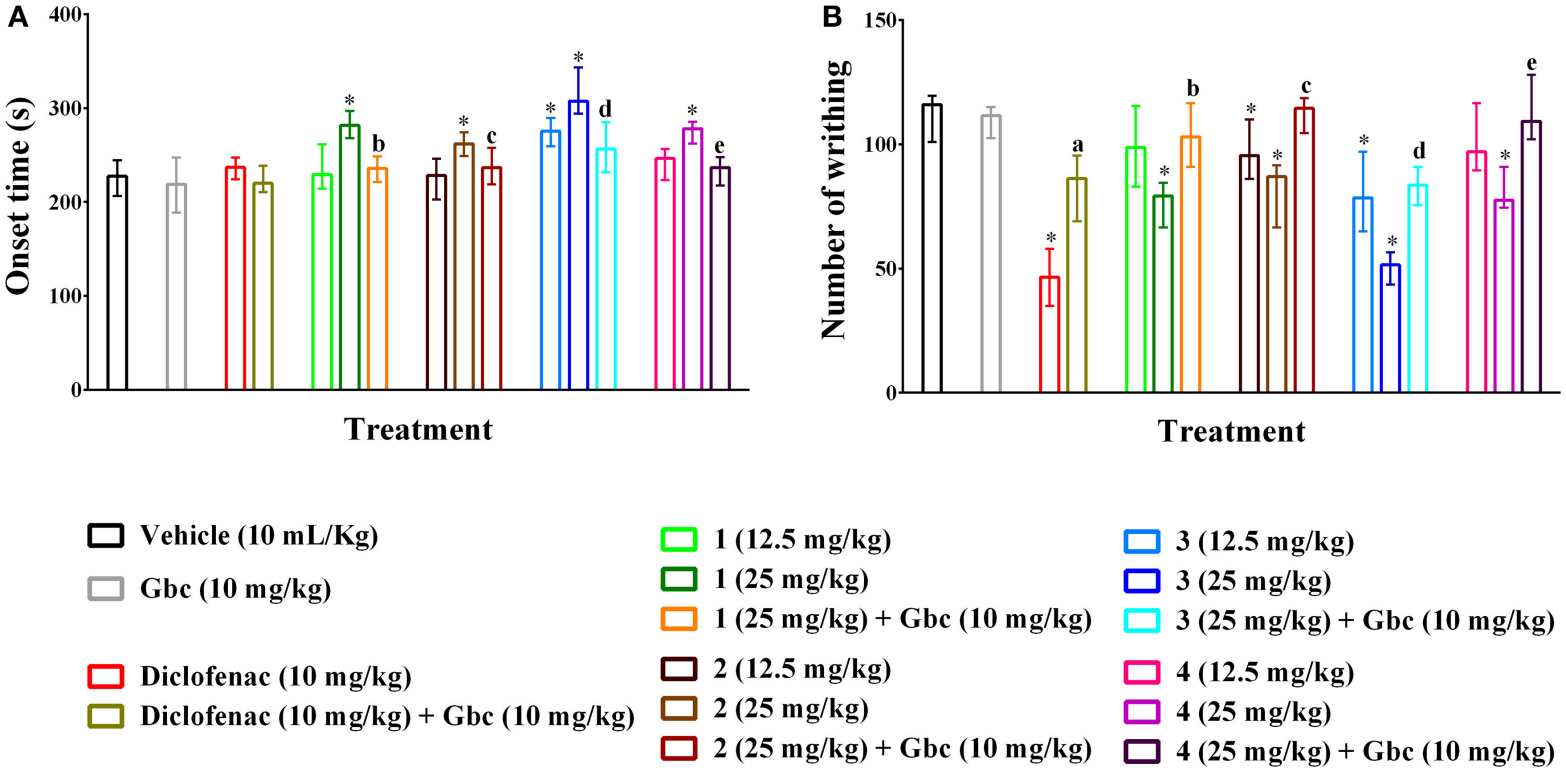
Effect of polymethoxyflavones (PMFs) of *N. plumbaginifolia* in acetic acid-induced writhing test. The panel shows effect on writhing onset **(A)**, total number of writhing **(B)**. Values are presented as median (*n* = 6) with range (min-max). **1** = 3,3′,5,6,7,8-hexamethoxy-4′,5′-methylenedioxyflavone; **2** = exoticin; **3** = 6,7,4′,5′-dimethylenedioxy-3,5,3′-trimethoxyflavone; **4** = 3,3′,4′,5,5′,8-hexamethoxy-6,7-methylenedioxyflavone, Gbc = glibenclamide. *p < 0.01 compared to control group; ^a,b,c,d,e^*p* < 0.01 compared to **1** (25 mg/kg), **2** (25 mg/kg), **3** (25 mg/kg), and **4** (25 mg/kg), respectively.

Compounds **1–4** increased writhing onset time (Figure 2A) as well as diminished the writhing episodes (Figure 2B) induced by acetic acid. The effects were significant (*p* < 0.01) for maximum experimental doses of compound **1**, **2** and **4** and all doses of compound **3** compared to control group. The onset time of writhing for compound **3** was found as 307.34 s (294.25–343.36 s) at the dose of 25 mg/kg b.w. which was longer than diclofenac 236.95 s (224.22–247.19 s). Compounds **1**, **2**, and **4** (25 mg/kg b.w.) could show maximum increase of onset time of writhing from 227.64 s (206.24–244.65 s) (showed by control group) to 281.62 s (268.20–296.93 s), 261.93s (248.92–274.38 s) and 278.17s (262.41–285.34 s) as well as reduction of total number of writhing from 116.00 (101.00–119.50) (induced by control group) to 79.20 (66.50–84.50), 87.00 (66.50–91.50) and 77.50 (74.50–91.00), respectively. Compound **3** caused the highest reduction of writhings [51.50 (43.50–56.50)] at the dose of 25 mg/kg b.w. than the other PMFs. Maximal of the effect was found for diclofenac [46.50 (35.00–58.00)]. The inhibition of total writhing episodes was dependent on dose and maximum inhibition of 59.77, 31.68, 24.59, 54.90, and 29.29% was seen for diclofenac and compounds **1–4**, respectively. In this experiment, glibenclamide (Gbc) treated mice could not produce any significant differences of writhing onset time [218.95s (188.47–247.32 s)] (Figure 2A) and writhing episodes [111.50 (102.50–115.00)] (Figure 2B) with respect to control group mice. However, pre-treatment of Gbc significantly (*p* < 0.01) diminished the effect of compounds **1–4** (25 mg/kg) on writhing protection by 9.96, 0.40, 28.02, and 7.41% respectively. The significant effect of diclofenac, experimental compounds **1–4** as well as pre-treatment of Gbc on writhing onset time and writhing at multiple time intervals are presented in Supplementary Tables 1, 2.

### Hot plate

**Figure 3:**
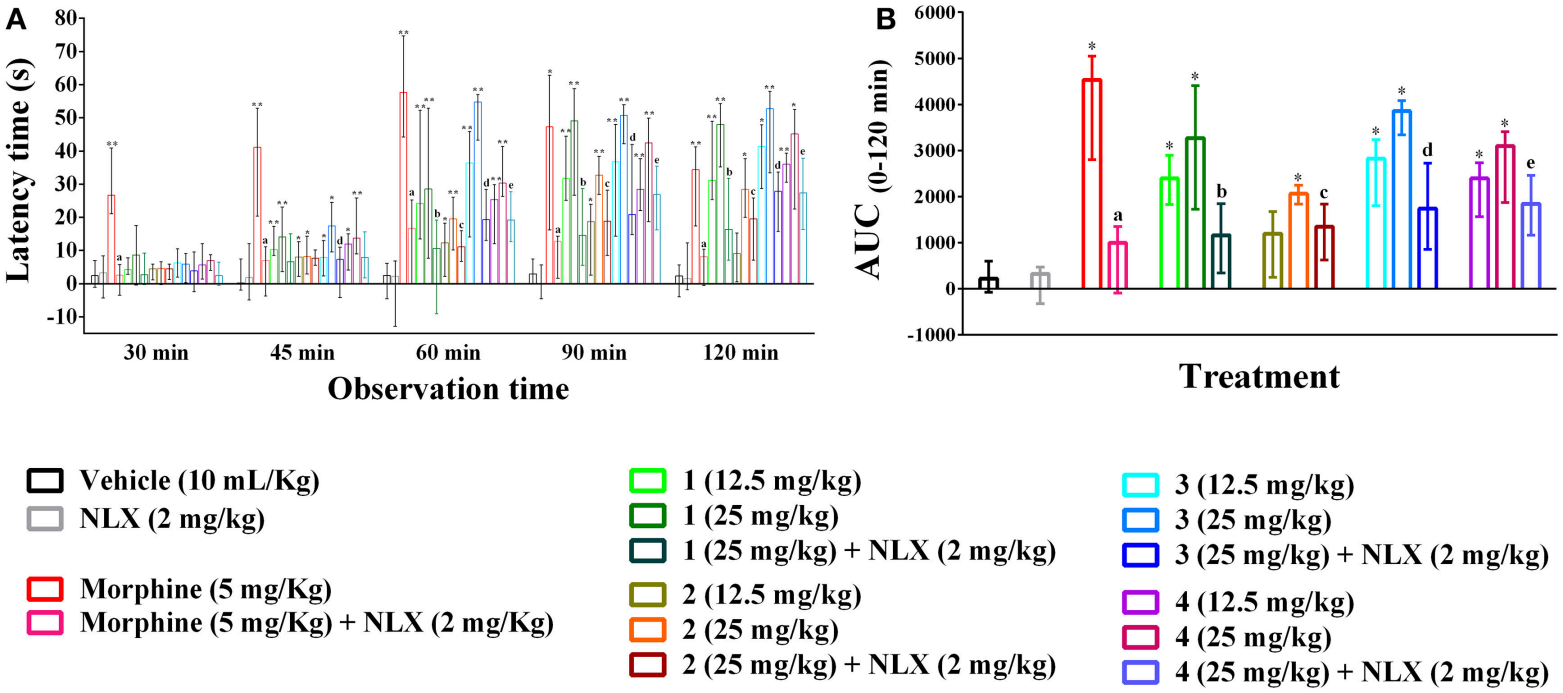
Effect of compounds **1–4**, morphine and pre-treatment of naloxone in hot plate test. **(A)** Maximal percent of analgesic effect (% MPE) of compounds **1–4**, morphine and antagonist effect of naloxone. **(B)** Area under the curve (AUC _0–120 min_) response of the compounds **1–4**, morphine and pre-treatment of naloxone. Values are presented as median (*n* = 6) with range (min-max). **1** = 3,3′,5,6,7,8-hexamethoxy-4′,5′-methylenedioxyflavone; **2** = exoticin; **3** = 6,7,4′,5′-dimethylenedioxy-3,5,3′-trimethoxyflavone; **4** = 3,3′,4′,5,5′,8-hexamethoxy-6,7-methylenedioxyflavone, NLX = naloxone. ^*^, ^**^p < 0.05 and *p* < 0.01, compared to control group, respectively. ^a,b,c,d,e^*p* < 0.05, compared to morphine (5 mg/kg), **1** (25 mg/kg), **2** (25 mg/kg), **3** (25 mg/kg), and **4** (25 mg/kg), respectively.

The hot plate experiment showed that orally treated mice with compounds **1–4** significantly (*p* < 0.01) produced analgesia against thermal threshold compared to control group (Figure 3A). The effect was dose reliant and significant from 45 min onwards for all the compounds. However, Compound **3** showed maximum thermal analgesia at the dose of 25 mg/kg b.w. at all the observation sessions compared to other experimental PMFs. Morphine (5 mg/kg b.w., i.p.) also exhibited the nociceptive protection effect over the experimental session and the effect was maximum by 26.69 (21.01–40.85) to 57.61 (44.22–74.64)% at the earlier experimental period (30–60 min). Compound **1** showed more potent effect than morphine at the dose of 25 mg/kg b.w. in later from 90 to 120 min by 50.74 (42.21–53.99) to 52.67 (33.38–58.03)%, respectively. The AUC response (Figure 3B) showed that morphine and compounds **1–4** significantly (*p* < 0.01) produced 20.62, 14.87, 10.20, 17.53, 15.49 times higher thermal protection compared to control group respectively. Naloxone pre-treatment reverted the antinociceptive action of compounds **1–4** and morphine significantly (*p* < 0.05), where it itself could not induce any significant differences in thermal protection with respect to control group. The effect of compounds **1–4**, morphine and naloxone pre-treatment on the latency time was also significant (*p* < 0.05) at different experimental periods as shown in Supplementary Table 3.

### Tail immersion

**Figure 4:**
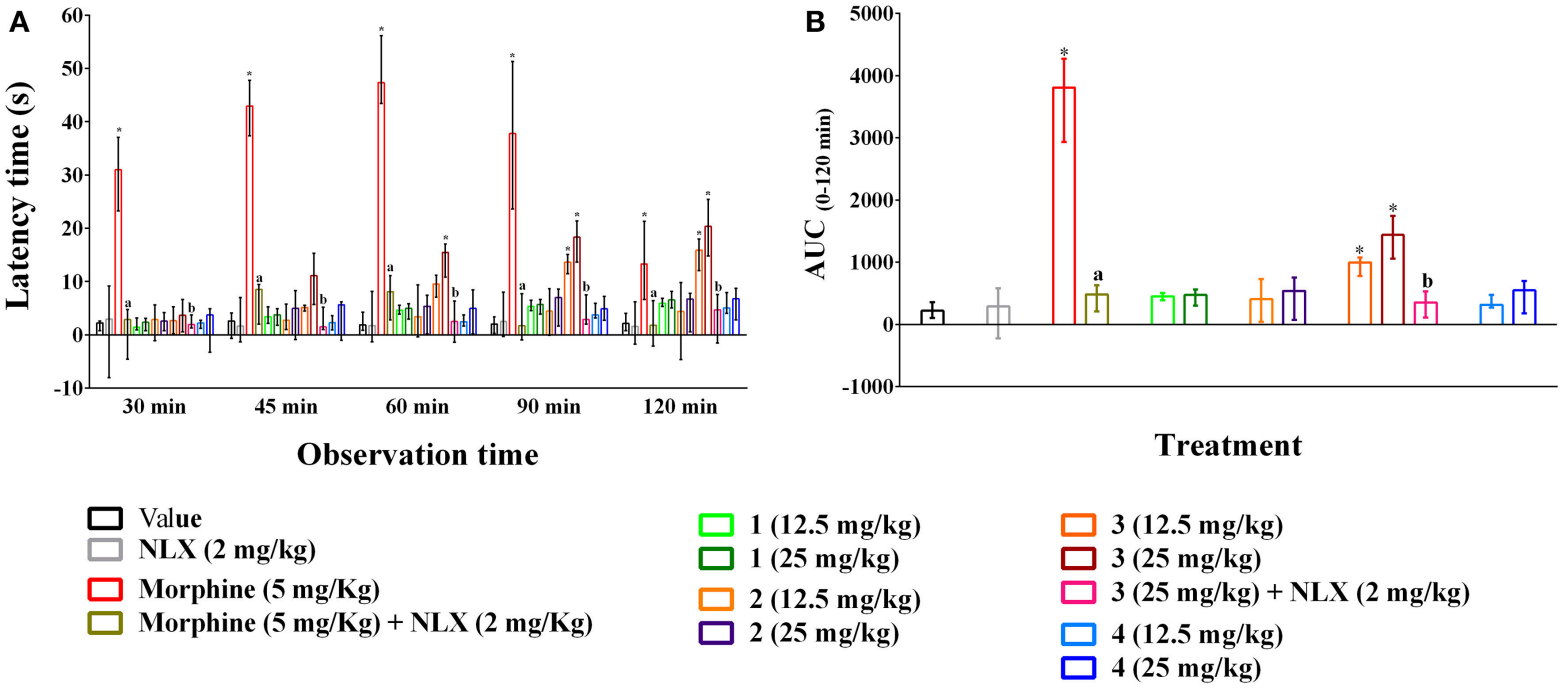
Effect of compounds **1–4**, morphine and pre-treatment of naloxone in tail immersion test. **(A)** maximal percent of analgesic effect (% MPE) of compounds **1–4**, morphine and antagonist effect of naloxone. **(B)** area under the curve (AUC _0−120__min_) response of the compounds **1–4**, morphine and pre-treatment of naloxone. Values are presented as median (*n* = 6) with range (min-max). **1** = 3,3′,5,6,7,8-hexamethoxy-4′,5′-methylenedioxyflavone; **2** = exoticin; **3** = 6,7,4′,5′-dimethylenedioxy-3,5,3′-trimethoxyflavone; **4** = 3,3′,4′,5,5′,8-hexamethoxy-6,7-methylenedioxyflavone, NLX = naloxone. ^*^p < 0.01, compared to control group. ^a,b^*p* < 0.01, compared to morphine (5 mg/kg) and **3** (25 mg/kg) respectively.

Oral ingestion of compounds **1–4** caused the thermal pain protection in tail immersion test (Figures 4A,B). However, only compound **3** and morphine could approach the statistical significance (*p* < 0.01). Compound **3** showed dose-dependent protection in thermally induced pain and the effect was statistically significant (*p* < 0.01) from 45 min onwards for all experimental doses. Morphine (5 mg/kg b.w., i.p.) exhibited significant reduction in thermal nociception for all observational sessions while the maximal effect was seen for compound **3** at the dosage of 25 mg/kg b.w. by 20.35 (14.80–25.45)% on 120 min than it [13.30 (6.65–21.34)%] (Figure 4A). The AUC_0−120_ min response demonstrated that thermal nociceptive protection by morphine and compound **3** at 12.5 and 25 mg/kg b.w. was 11.91, 3.00, and 4.87 times greater and statistically significant (*p* < 0.01) with respect to control group respectively (Figure 4B). Naloxone treated animals could not produce any significant effect compared to control animals. Naloxone pre-treatment reversed the percent analgesic as well as AUC_0−120__min_ effect of morphine and compound **3** (Figures 4A,B). Morphine and compound **3** and pre-treatment of naloxone also caused significant differences (*p* < 0.01) in thermal latency time at multiple observational periods compared to control group as shown in Supplementary Table 4.

### Formalin-induced nociception and paw edema

The treatment of experimental PMFs **1–4** (p.o.) and morphine (i.p.) diminished the formalin induced early and late phase nociceptive responses as shown in Table 2. The effect was significant (*p* < 0.05) for both phases by morphine and experimental PMFs (**1–4**). All test samples produced the highest protection of nociception at the maximum dose (25 mg/kg b.w.) and their effect was dose dependent. The maximum inhibition was produced by morphine (5 mg/kg b.w.) where the effect of compound **3** was higher compared to other test samples at both phases. In addition, the inhibition of nociceptive responses was stronger at late phase for morphine and all test compounds. The data for time vs. nociceptive effects of morphine, and compounds **1–4** treatments on formalin-induced nociception are depicted on Supplementary Figures 1A–D. Treatment of morphine and compound **1**, **3** and **4** could also significantly (*p* < 0.05) reduce the formalin-induced paw edema at all experimental doses (Table 2). Treatments with exoticin (**2**) could not approach the statistical significance of the effect. The maximal inhibition of edematogenic response was exhibited by morphine (58.48%) where compound **4** showed highest response (43.25%) with respect to other PMFs at the dose of 25 mg/kg b.w.

**Table 2:** Effect of morphine, compounds **1–4** in formalin-induced nociception and paw edema test.

**Treatment**	**Dose (mg/kg)**	**Total responses time (s)**				**Paw edema (Δ in mm) after 5 h**	**% inhibition**
		**Early phase (0–10 min)**	**% inhibition**	**Late phase (11–60 min)**	**% inhibition**		
Vehicle	–	209.21 (165.55–225.36)	–	471.10 (395.21–587.97)	–	1.45 (1.32–1.65)	–
Morphine	5	41.70^**^ (38.01–45.10)	80.08	17.99^**^ (10.41–24.40)	96.18	0.60^**^ (0.33–0.91)	58.48
**1**	12.5	190.17 (170.35–205.85)	9.10	433.05 (366.27–484.51)	8.08	1.08^**^ (0.83–1.26)	25.61
**1**	25	146.95^**^ (134.69–161.44)	29.76	322.66^**^ (271.24–346.27)	31.51	1.05^**^ (0.86–1.10)	27.34
**2**	12.5	209.80 (189.05–231.63)	−0.28	443.17 (379.02–502.71)	5.93	1.23 (1.05–1.63)	14.88
**2**	25	182.83^*^ (165.36–204.96)	12.61	382.31^*^ (337.14–443.45)	18.15	1.19 (0.97–1.64)	17.65
**3**	12.5	135.65^*^ (116.81–171.60)	35.16	247.69^*^ (160.92–267.41)	47.42	0.91^**^ (0.84–1.14)	37.02
**3**	25	86.67^**^ (73.53–113.55)	58.57	160.45^**^ (141.55–226.59)	65.94	0.83^**^ (0.67–1.04)	42.91
**4**	12.5	170.57^*^ (157.06–186.42)	18.47	357.35^*^ (269.52–437.84)	24.15	0.99^**^ (0.92–1.06)	31.49
**4**	25	132.12^**^ (116.08–149.21)	36.85	236.85^**^ (186.05–392.11)	49.72	0.82^*^ (0.48–1.71)	43.25

*Values are presented as median (n = 6) with range (min-max). **1** = 3,3′,5,6,7,8-hexamethoxy-4′,5′-methylenedioxyflavone; **2** = exoticin; **3** = 6,7,4′,5′-dimethylenedioxy-3,5,3′-trimethoxyflavone; **4** = 3,3′,4′,5,5′,8-hexamethoxy-6,7-methylenedioxyflavone*.

*, ** *p < 0.05 and p < 0.01, compared to control group, respectively*.

### Carrageenan-induced paw edema

The subplantar injection of carrageenan to the control group mice caused gradual increase in paw edema from 1 to 5 h, which decreased at 6 h as shown in Table 3. Mice treated with experimental PMF **1**, **3** and **4** at all test doses caused significant (*p* < 0.05) reduction of paw edema from 3 to 6 h compared to control group. Compound **3** at the dose of 25 mg/kg b.w., p.o. and diclofenac (10 mg/kg b.w., i.p.) exhibited significant reduction as well as percent inhibition of paw edema from 1 h to end of the experimental period. PGE2 content (equivalent to OD) of diclofenac and PMF **1**, **3** and **4** treated mice were significantly (*p* < 0.01) less than thecontrol group. Exoticin (**2**) could neither inhibit edematogenic syndrome nor PGE2 content by any of the experimental dose. The inhibition of paw edema throughout the entire experimental time and reduction of PGE2 content was maximum by diclofenac where compound **3** showed highest at 25 mg/kg b.w. compared to other tested PMFs (Table 3). The result also showed that there was dose effect for all tested compounds.

**Table 3:** Effect of morphine, compounds **1–4** in carrageenan-induced paw edema test.

**Treatment**	**Dose (mg/kg)**	**Paw edema thickness (Δ) in mm [% inhibition]**							**PGE2 content (equivalent to OD)**
		**0 h**	**1 h**	**2 h**	**3 h**	**4 h**	**5 h**	**6 h**	
Vehicle	–	1.21 (1.14–1.35)	1.84 (1.38–2.00)	1.93 (1.80–2.11)	2.11 (2.00–2.46)	2.21 (2.07–2.67)	2.26 (2.14–2.64)	2.01 (1.94–2.40)	9.66 (7.12–11.18)
Diclofenac	10	1.26 (1.04–1.42)	1.11^**^ (0.78–1.21) [39.95]	1.14^**^ (0.86–1.29) [41.04]	1.09^**^ (0.96–1.43) [48.22]	1.06^**^ (0.97–1.32) [52.15]	1.05^**^ (0.74–1.30) [53.66]	0.82^**^ (0.35–1.12) [59.90]	2.13^**^ (1.91–2.75)
**1**	12.5	1.20 (1.15–1.29)	1.60 (1.30–1.84) [13.04]	1.70 (1.35–1.98) [11.69]	1.79^*^ (1.42–2.18) [15.20]	1.82^**^ (1.54–2.00) [17.46]	1.82^**^ (1.52–2.08) [19.29]	1.62^*^ (1.29–2.00) [19.65]	6.09^*^ (5.53–7.03)
**1**	25	1.25(1.15–1.43)	1.58 (1.36–1.73) [14.40]	1.67^**^ (1.48–1.79) [13.25]	1.74^**^ (1.53–1.83) [17.58]	1.74^**^ (1.53–2.01) [21.32]	1.77^**^ (1.56–2.03) [21.51]	1.50^**^ (1.35–1.84) [25.62]	5.26^**^ (4.34–6.00)
**2**	12.5	1.22 (1.18–1.32)	1.67 (1.55–1.89) [9.24]	1.82 (1.60–2.13) [5.45]	1.93 (1.87–2.46) [8.31]	2.08 (1.86–2.70) [5.90]	2.25 (2.04–2.55) [0.22]	2.06 (1.73–2.30) [−2.49]	9.31 (7.46–11.24)
**2**	25	1.17 (1.03–1.43)	1.78 (1.25–2.01) [3.53]	1.77 (1.42–2.16) [8.05]	1.86 (1.56–2.49) [11.88]	2.05 (1.49–2.59) [7.26]	2.16 (1.54–2.65) [4.21]	1.90 (1.36–2.43) [5.47]	8.51 (7.39–10.23)
**3**	12.5	1.23 (1.21–1.30)	1.50^*^ (1.36–1.57) [18.48]	1.62^**^ (1.43–1.73) [16.10]	1.74^**^ (1.53–2.01) [17.34]	1.64^**^ (1.57–2.17) [25.62]	1.69^**^ (1.51–1.79) [25.28]	1.42^**^ (1.22–1.56) [29.60]	5.17^**^ (3.89–6.13)
**3**	25	1.24 (1.03–1.58)	1.33* (1.08–1.72) [27.99]	1.44^**^ (1.16–1.85) [25.19]	1.66^**^ (1.23–1.84) [21.14]	1.55^**^ (1.22–2.00) [29.71]	1.53^**^ (1.25–1.91) [32.15]	1.25^**^ (1.01–1.74) [37.81]	4.07^**^ (3.25–4.99)
**4**	12.5	1.37 (0.91–1.46)	1.55^*^ (1.31–1.70) [15.76]	1.65^**^ (1.40–1.79) [14.55]	1.77^**^ (1.53–1.99) [15.91]	1.77^**^ (1.64–2.06) [19.55]	1.80^**^ (1.61–2.02) [20.18]	1.52^**^ (1.32–1.70) [24.38]	5.52^**^ (4.94–6.99)
**4**	25	1.24 (1.05–1.38)	1.45^*^ (1.24–1.76) [21.20]	1.54^**^ (1.30–1.93) [20.26]	1.63^**^ (1.37–2.15) [22.80]	1.65^**^ (1.42–2.19) [25.40]	1.70^**^ (1.40–2.27) [24.83]	1.44^**^ (1.15–1.90) [28.36]	4.54^**^ (3.97–6.18)

*Values are presented as median (n = 6) with range (min-max). **1** = 3,3′,5,6,7,8-hexamethoxy-4′,5′-methylenedioxyflavone; **2** = exoticin; **3** = 6,7,4′,5′-dimethylenedioxy-3,5,3′-trimethoxyflavone; **4** = 3,3′,4′,5,5′,8-hexamethoxy-6,7-methylenedioxyflavone*.

*, ** *p < 0.05 and p < 0.01, compared to control group, respectively*.

### Hole cross

The treatment of experimental compounds **2**, **3** (p.o) and diazepam (i.p.) caused the decrease of movement of mice in the hole cross test compared to control group mice (Table 4). However, the effect was significant (*p* < 0.05) for compound **2** and **3** at the dose of 25 mg/kg b.w. Diazepam (1 mg/kg b.w.) and compound **2** (25 mg/kg b.w.) administration produced significant inhibition of movement at all experimental periods where the effect was maximum by exoticin at 30 min and from 90 to 120 min with respect to other experimental compounds. Compound **3** could cause significant inhibition during the 60–90 min interval. In contrast, compound **1** and **4** could not produce any significant differences in effect at any tested dose compared to control group (Table 4).

**Table 4:** Effect of diazepam, compounds **1–4** in hole cross test.

**Treatment**	**Dose (mg/kg)**	**Number of hole crossed [% inhibition]**				
		**0 min**	**30 min**	**60 min**	**90 min**	**120 min**
Vehicle	–	10.50 (8–13)	10.00 (7–12)	8.50 (8–12)	6.00 (5–10)	5.00 (4–8)
Diazepam	1	10.00 (8–13)	7.00^*^ (2–8) [30.00]	5^*^ (3–6) [41.18]	3.00^*^ (2–4) [50.00]	2.50^*^ (2–3) [50.00]
**1**	12.5	9.00 (8–12)	8.50 (5–12) [15.00]	8.50 (6–12) [0.00]	5.50 (2–9) [8.33]	5.00 (2–8) [0.00]
**1**	25	10.50 (9–14)	8.00 (6–13) [20.00]	7.50 (5–11) [11.76]	5.00 (3–7) [16.67]	4.50 (2–8) [10.00]
**2**	12.5	11.00 (10–13)	8.00 (7–9) [23.53]	6.50 (5–11) [20.00]	4.50 (3–7) [25.00]	4.00 (2–6) [20.00]
**2**	25	10.50 (6–12)	6.00^*^ (3–7) [40.00]	5.50^*^ (3–6) [35.29]	2.50^*^ (1–4) [58.33]	2.50^*^ (1–4) [50.00]
**3**	12.5	10.50 (7–12)	9.50 (3–10) [5.00]	6.00 (3–8) [29.41]	4.00 (2–6) [33.33]	4.00 (2–9) [20.00]
**3**	25	12 (9–13)	7.50 (5–10) [25.00]	4.50^*^ (3–7) [47.06]	3.00^*^ (2–5) [50.00]	4.00 (2–6) [20.00]
**4**	12.5	11.50 (9–14)	8.50 (6–10) [15.00]	7.50 (6–9) [11.76]	5.00 (2–8) [16.67]	3.50 (2–10) [30.00]
**4**	25	10.50 (9–13)	8.00 (5–10) [20.00]	6.00 (4–11) [29.41]	4.50 (3–7) [25.00]	4.00 (3–6) [20.00]

*Values are presented as median (n = 6) with range (min-max). **1** = 3,3′,5,6,7,8-hexamethoxy-4′,5′-methylenedioxyflavone; **2** = exoticin; **3** = 6,7,4′,5′-dimethylenedioxy-3,5,3′-trimethoxyflavone; **4** = 3,3′,4′,5,5′,8-hexamethoxy-6,7-methylenedioxyflavone*.

* *p < 0.05, compared to control group*.

### Open field

**Figure 5:**
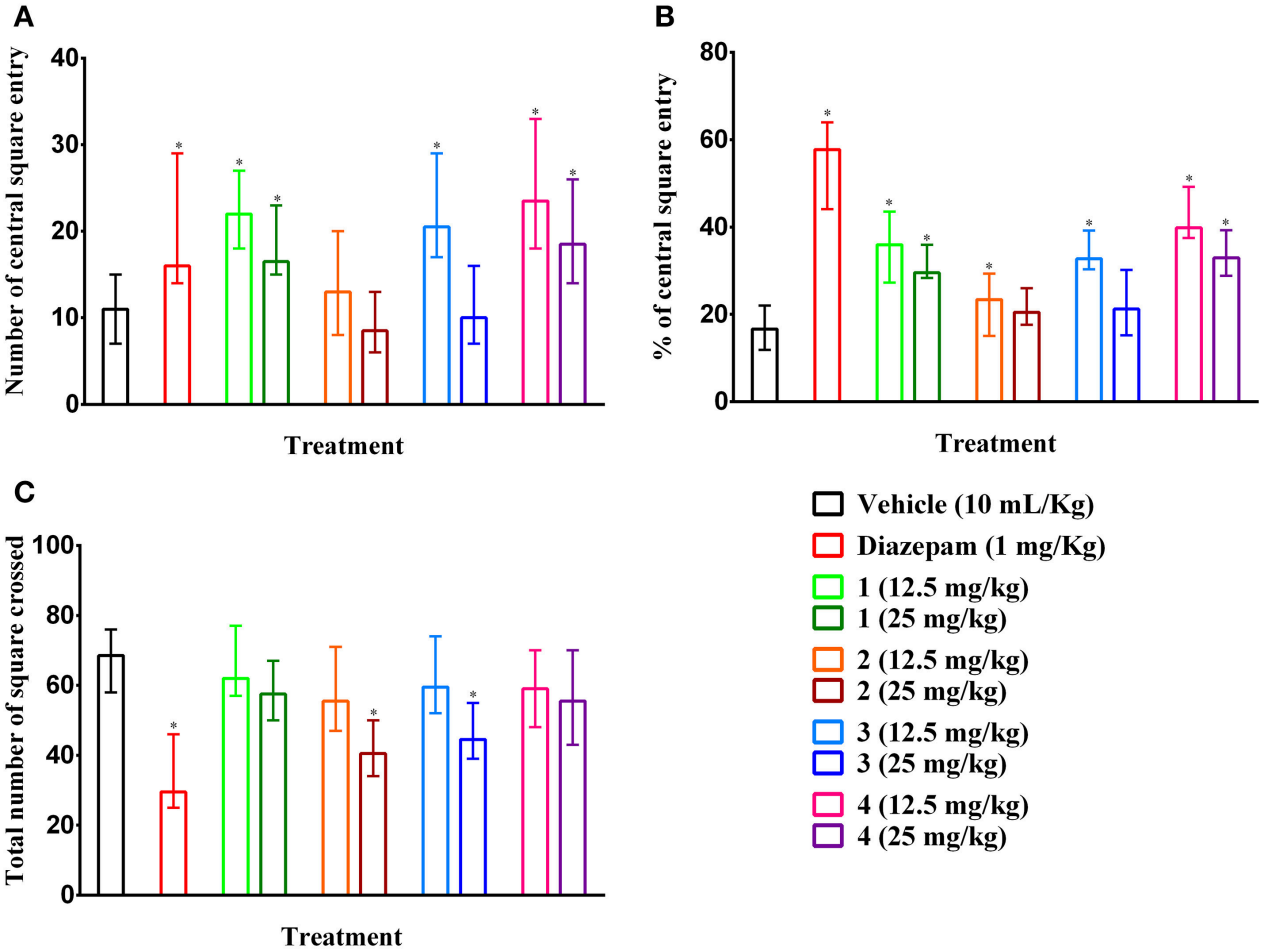
Ambulatory effect of compounds **1–4** and diazepam in open field test. **(A–C)** Represents effect of PMFs **1–4** and diazepam treatment on total number of central squares entries, percent of central squares entries and total number of squares crossed in open field, respectively. Values are presented as median (*n* = 6) with range (min-max). **1** = 3,3′,5,6,7,8-hexamethoxy-4′,5′-methylenedioxyflavone; **2** = exoticin; **3** = 6,7,4′,5′-dimethylenedioxy-3,5,3′-trimethoxyflavone; **4** = 3,3′,4′,5,5′,8-hexamethoxy-6,7-methylenedioxyflavone. ^*^p < 0.05, compared to control group.

Compounds **2** and **3** (25 mg/kg b.w., p.o.) and diazepam (1 mg/kg b.w., i.p.) treated mice showed significant (*p* < 0.05) reduction in the number of square crossings 40.50 (34–50), 44.50 (39–55), 29.50 (25–46) by 40.88, 35.04, and 56.93 %, respectively compared to control group mice [68.50 (58–76)] (Figure 5C). Any significant alteration of the locomotor action was absent for compound **1** and **4** at any tested dose. The plant PMF **1**, **3**, **4** and diazepam caused significant (*p* < 0.05) increase of entries in central squares (Figure 5A). However, the effect was reduced due to the increase in the dose of experimental PMFs. Exoticin (**2**) treated mice did not exhibit significant increase in central square entries at 12.5 [13.00 (8–20)] and 25 mg/kg b.w. [8.50 (6–13)] compared to control group mice [11.00 (7–15)]. Compounds **1**, **3**, and **4** (12.5 mg/kg b.w.) and diazepam treatment showed highest number of entries of central squares [22.00 (18–27), 20.50 (17–29), 23.50 (18–33), and 16.00 (14–29)] (Figure 5A) by 35.95 (27.27–43.55), 32.74 (30.36–39.19), 39.83 (37.50–49.25), and 57.75 (44.12–64.00)%, respectively (Figure 5B). In addition, percent of entries of central squares by the treatments was also significant (*p* < 0.05) compared to control group [16.67 (11.84–22.06)%]. Diazepam treatment caused maximum inhibition of a total number of square entries but an increase of percent of entries of central squares.

### Elevated plus maze

**Figure 6:**
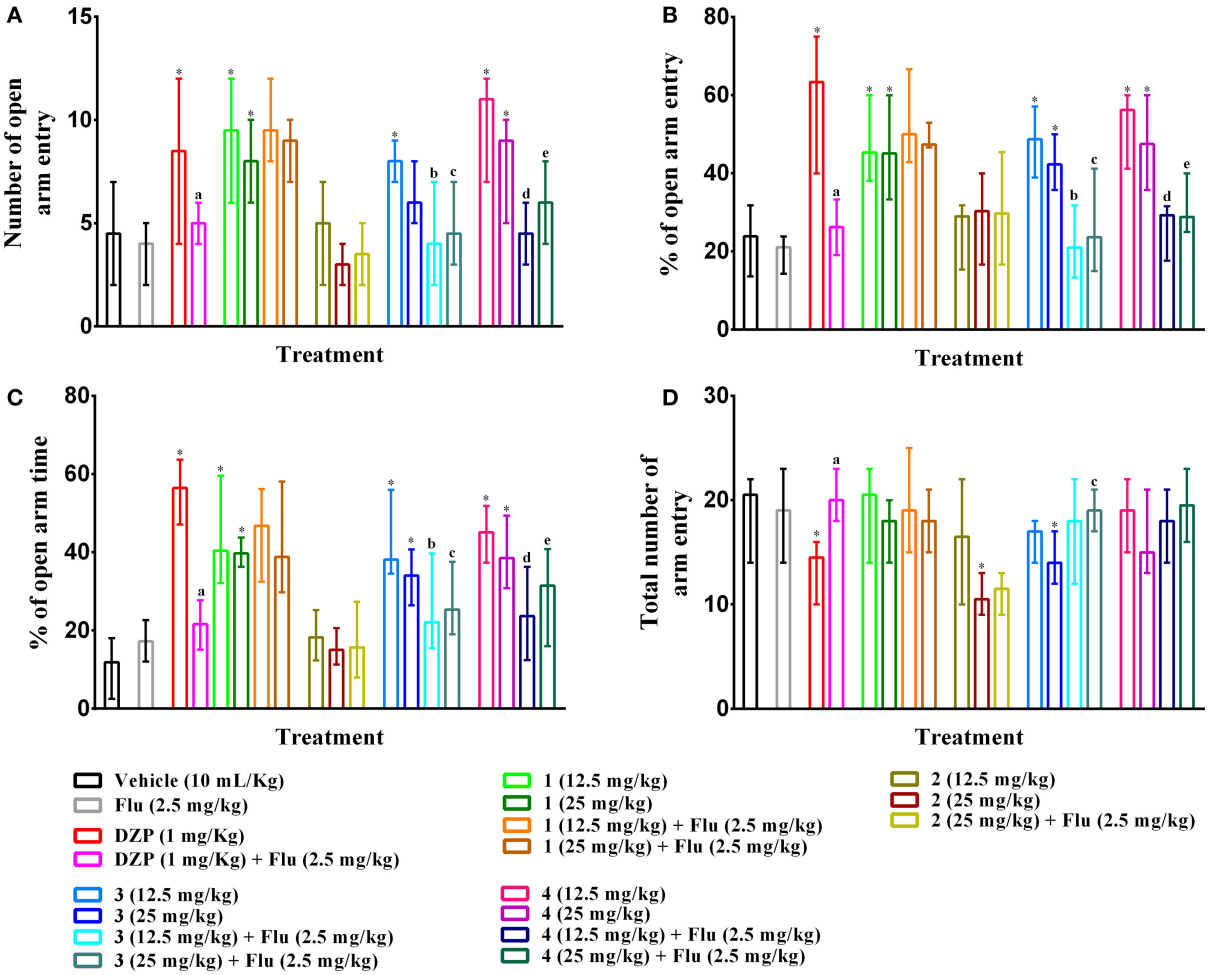
Effect of compounds **1–4**, diazepam and pre-treatment of flumazenil in elevated plus maze test. The illustrations represent effect of PMFs **1–4** and diazepam treatment on **(A)** total number of open arms entries, **(B)** percent of central squares entries, **(C)** percent of open arm time, and **(D)** total number arm entries in elevated plus maze. Values are presented as median (*n* = 6) with range (min-max). **1** = 3,3′,5,6,7,8-hexamethoxy-4′,5′-methylenedioxyflavone; **2** = exoticin; **3** = 6,7,4′,5′-dimethylenedioxy-3,5,3′-trimethoxyflavone; **4** = 3,3′,4′,5,5′,8-hexamethoxy-6,7-methylenedioxyflavone, Flu, flumazenil. ^*^p < 0.05, compared to control group. ^a,b,c,d,e^*p* < 0.05, compared to diazepam (1 mg/kg), **3** (12.5 mg/kg), **3** (25 mg/kg), **4** (12.5 mg/kg), and **4** (25 mg/kg) respectively.

As illustrated in Figures 6A–C, PMF **1**, **3**, **4** (p,o) and diazepam (i.p.) treatment significantly (*p* < 0.05) increased the entries, percent entries as well as exploration time in open arms effects of mice with respect to control group mice in elevated plus maze test. The results also showed that effects of experimental PMFs were decreased for the increased of doses. Compound **1**, **3**, **4** (12.5 mg/kg b.w.) and diazepam produced maximum number of open arms entries [9.50 (6–12), 8.00 (7–9), 11 (7–12) and 8.50 (4–12)], (Figure 6A), by 45.34 (38.10–60.00), 48.69 (38.89–57.14), 56.22 (41.18–60.00) and 63.33 (40.00–75.00)%, (Figure 6B), respectively where vehicle treated mice showed 4.50 (2–7) by 23.89 (13.64–31.82)%. Experimental animals exhibited highest open arms spent time at 12.5 mg/kg b.w. of PMF **1** [40.39 (32.13–59.59)%], **3** [38.13 (34.58–55.99)%] and **4** [45.11 (37.38–51.87)%] whereas diazepam treatment (1 mg/kg b.w.) demonstrated maximum activity [56.46 (47.12–68.73)%], (Figure 6C). Compound **2**, **3** (25 mg/kg b.w.) and diazepam significantly (*p* < 0.05) decreased the total arms entries [10.50 (9–13), 14.00 (12–17), and 14.50 (10–16), respectively] compared to control group [20.50 (14–22)], (Figure 6D). The pre-treatment of flumazenil significantly (*p* < 0.05) diminished the effects produced by diazepam, compound **3** and **4** (Figures 6A–D). However, it could not produce any significant entries [4.00 (2–5)], percent entries [21.05 (14.29–23.81)] as well as spent time [17.21 (12.04–22.61)%] in the open arms and total arm entries [19.00 (14–23)] responses of mice by itself with respect to control group. Flumazenil could not alter the activities of compound **1** and **2** treated groups.

## Discussion

The results of present study revealed antinociceptive activity of polymethoxyflavone (PMF)- 3,3′,5,6,7,8-hexamethoxy-4′,5′-methylenedioxyflavone (**1**), exoticin (**2**), 6,7,4′,5′-dimethylenedioxy-3,5,3′-trimethoxyflavone (**3**), 3,3′,4′,5,5′,8-hexamethoxy-6,7-methylenedioxyflavone (**4**) of *N. plumbaginifolia* in different central and inflammatory models of pain. Neuropharmacological studies of the compounds revealed the possibilities of their anti-anxiety effect. Furthermore, investigation of possible mechanisms of these effects created an insight on their pharmacological activities.

The PMFs of *N. plumbaginifolia* significantly (*p* < 0.01) delayed the onset as well as caused the inhibition of writhing episodes (Figures 2A,B) in the acetic acid-induced writhing test. This test is a reliable method for the evaluation of central and peripheral analgesic effect of new agents (Le Bars et al., 2001). Administration of acetic acid (i.p.) causes liberation of cyclooxygenase (COX), prostaglandins (PGs), lipoxygenase (LOX), histamine, serotonin, bradykinin, and cytokinin (TNF-α, IL-8, IL-1β), in the tissue of visceral fluid. These inflammatory mediators disrupt as well as increase the permeability of blood brain barrier (BBB) and excite primary afferent nociceptors by entering into the dorsal horn of central nervous system (CNS). This results in a pathological condition of pain, which is characterized as writhing (Ikeda et al., 2001; Radu et al., 2013). Therefore, it could be suggested that the PMFs diminished the release of the acid-induced endogenous inflammatory mediators. The delayed writhing onset by the PMFs indicates their interruption in nociceptive signals transduction to primary afferent nociceptors. In addition, attenuation in the numbers of acetic writhing episodes, delayed onset time of writhing, might be attributed to the down-regulation of the inflammatory cytokines TNF-α, IL-1β, and IL-6 proteins (Yin et al., 2016).

PMFs **1–4** demonstrated significant protection of thermally-induced pain in the hot plate test (Figures 3A,B). However, thermal analgesia by compound **3** was significant only in the tail immersion test (Figures 4A,B). These methods are employed for the evaluation of centrally acting drugs and can be distinguished based on their pathway of induction of nociception. The hot plate test is selective for the supraspinally mediated nociception whereas tail immersion in hot water induces spinally mediated nociception (Chapman et al., 1985). Opioid agents involve spinal (μ_1_, δ_1_, κ_3_) and supraspinal (μ_1_, δ_1_, σ_2_, κ_3_) receptors for their analgesic action (Hosseinzadeh et al., 2002; Jinsmaa et al., 2004, 2005). Therefore, the results indicated that inhibition of nociception by compound **3** could be associated with both spinal and supraspinal opioid receptors whereas compound **1**, **2** and **4** could involve supraspinal opioid receptors.

Formalin administration in the subplantar region of paw elicits biphasic nociceptive pain. In the first phase (0–10 min), it causes neurogenic pain by the direct excitation of unmyelinated and myelinated sensory afferent fibers, especially C-fibers and releases substance P and bradykinin. The second phase (11–50 min) causes inflammatory pain by the release of excitatory mediators including histamine, bradykinin, serotonin, prostaglandins (PGs) in the peripheral tissues and disrupting the neuronal function of the central dorsal horn. The release of tissue inflammatory mediators also results in paw edema, which peaks at 5 h and could be inhibited by the supraspinal inputs of CNS (Wheeler-Aceto and Cowan, 1991; Tjølsen et al., 1992; França et al., 2001; Campos et al., 2002; Dai et al., 2002). The formalin-induced paw edema and licking/biting response of paw may have related to the release of inflammatory cytokines, p-CASP6, a-CASP6, TNF-α, IL-1β, and IL-6 proteins (Yin et al., 2016). Therefore, it could be suggested that the test compounds have downregulated the inflammatory cytokines. It has been reported that peripherally acting drugs like acetyl salicylic acid, naproxen, indomethacin inhibit the release of histamine, bradykinin, serotonin, prostaglandins (PGs) and attenuate the second phase pain whereas centrally acting drugs suppresses the nociception of both phases in formalin test (Tjølsen et al., 1992; França et al., 2001). PMFs **1**, **3**, **4** significantly (*p* < 0.01) reduced the formalin-induced nociceptive responses of both phases and the effect was more pronounced in the inflammatory phase. In addition, they have caused a significant (*p* < 0.05) reduction of formalin-induced paw edema (Table 2), which suggested involvement of supraspinal systems in their antinociceptive action. These results indicated the central antinociceptive as well as potential anti-inflammatory effects of the PMFs.

Carrageenan-induced paw edema test is widely used in the evaluation of the anti-edematogenic effect of the experimental compound. Subplantar injection of carrageenan produces a biphasic edematogenic response where serotonin, histamine, and kinins are released in the first phase (0–1 h), whereas the final phase (1–6 h) is associated with the release of prostaglandins (PGs), particularly of their E series by the activation of cyclooxygenase (COX) in tissues. Kinins provide the continuity between these inflammatory phases (Morris, 2003; Mothana, 2011). Inducible nitrogen oxide synthase (iNOS) might also be involved in the formation of carrageenan-induced paw edema (Salvemini et al., 1996; Mortada et al., 2017). The findings of the present study revealed that PMFs **1**, **3**, and **4** could significantly reduce carrageenan-induced paw edema (Table 3). Therefore, a significant decrease of both first and second phase edematogenic response by compound **3** could be due to the inhibition of release of serotonin, histamine, and PGs or downregulation of iNOS production. On the other hand, compound **3** and **4** could significantly inhibit the second phase edema suggesting their suppression of release of COX products as well as PGs. It has been found that PGE2 plays a major role in hyperalgesia, IL-6 production and tissue edema at sites of inflammation (Portanova et al., 1996). The significant (*p* < 0.01) reduction of PGE2 content by the plant PMF **1**, **3**, and **4** (Table 3) could be attributed to their anti-edematogenic as well as anti-inflammatory effect.

Previous studies reported that mono and polymethoxyflavones (PMFs) exert opioid mediated antinociceptive effect (Thirugnanasambantham et al., 1993; Pandurangan et al., 2014; Nadipelly et al., 2016). Structure activity relationships study revealed that the presence of methoxy group (-OCH_3_) at 5 or 7 positions on flavone moiety could induce opioid mediated analgesia (Thirugnanasambantham et al., 1993). The experimental PMFs of present study also comprises –OCH_3_ group at 5 and/or 7 positions. Moreover, results of the present investigation demonstrated that naloxone pre-treatment diminished the thermal analgesia of PMFs **1–4** in the hotplate or tail immersion test (Figures 3, 4). This confirmed the association of opioid system in the antinociceptive effect of the plant PMFs. The results also suggested that the effect of PMF **1** and **4** involved supraspinal, where PMF **3** involved both spinal and supraspinal opioid receptors.

Drugs acting on the spinal and supraspinal systems (e.g., morphine, diazoxide) may involve the ATP sensitive K^+^ channel (K_ATP_) for their antinociceptive effect. In addition, antinociceptive effect of diclofenac, ketorolac involves opening of K_ATP_ by increasing the intracellular cyclic guanosine monophosphate (cGMP) level in tissues. Activation of NO-cGMP pathway causes the opening of K_ATP_ followed by an effluxof K^+^ ion, membrane re- or hyper-polarization and reduces the membrane as well as cellular excitability (Lawson, 1996; Ocaña et al., 2004). Glibenclamide has been reported to selectively block the ATP sensitive K+ channels without affecting voltage gated and Ca^2+^ activated K^+^ channels (Alves and Duarte, 2002; Jesse et al., 2007). Results of the current study exhibited that the effects of PMFs **1–4** were significantly reversed by glibenclamide (Figures 2A,B). Thus, it can be suggested that the PMFs **1–4** might involve opening of K_ATP_ system followed by reduction of membrane and cellular excitability for their antinociceptive effect.

Oral treatment of experimental doses did not exert any toxicity, abnormalities of the organ or cause the death of animals, which indicated that the selected test doses were safe for the study. The neurobehavioral study showed that increase in the dose of the experimental PMFs caused a reduction in locomotor activity, whereas compounds **2** and **3** produced a significant (*p* < 0.05) reduction at a maximal experimental dose (25 mg/kg b.w.) in the hole cross test (Table 4). The effect was also reflected by diazepam and in the open field test (Figures 5A–C). However, compound **3** increased the number as well as the percent of central squares entries in the open field. As shown by the results, compounds **1** and **4** also increased the number as well as the percent of central squares entries in the open field but did not cause any significant alteration of the locomotor activity. It has been reported that anxiolytic drugs such as benzodiazepines and 5-HT_1A_ agonists increase central and percent of squares entries of mice on open field (Prut and Belzung, 2003). The results indicated that compounds **1**, **3**, and **4** could possess anxiolytic effect. Therefore, the plant isolates were subjected to evaluate anxiolytic activity using elevated plus maze test.

Elevated plus maze is a commonly applied method for the evaluation of anxiolytic activity of new agents (Dawson and Tricklebank, 1995) and it is validated for both rodents and mice (Pellow et al., 1985; Lister, 1987). The apparatus contains two open and closed arms. Animals show extreme aversion to exploring in the open arms. The indexes of anxiety- exploration time and number of entries in open arms are sensitive to the drugs that act on benzodiazepine as well as GABA_A_ receptor sites. These drugs increase the percent of open arm frequencies as well as exploration time and decreases total arm entries (Pellow et al., 1985; Griebel et al., 1996). The results of the experiment showed that PMFs **1**, **3**, **4** and diazepam significantly increased the percent of exploration time and frequency of entries in open arms (Figures 6B,C). These effects could be attributed to their anxiolytic activity. However, compounds **1** and **4** did not cause any significant reduction of total number of entries at the experimental doses, where compound **3** at the maximal dose (25 mg/kg) and diazepam produced significant (*p* < 0.05) reductions (Figure 6D). Flumazenil, a selective benzodiazepine antagonist of GABA_A_ receptor, significantly (*p* < 0.05) diminished the effects of compounds **3** and **4**. This confirmed the involvement of GABA_A_ receptor in their anxiolytic activity. The results showed that flumazenil could not attenuate the anxiolytic effect of PMF **1**, suggesting a different mechanism of action. Experimental results also demonstrated that an increase of dose reduced the anxiolytic effect of the PMFs. Drugs acting on benzodiazepine system has been reported to significantly decrease the locomotor function (Prut and Belzung, 2003). The effects could be due to the reduction of locomotor activity which are evident in the hole cross (Table 4) and open field test (Figure 5) results.

Overall, applications of *N. plumbaginifolia* in the treatment of different painful conditions are evident in traditional systems of medicine and analgesic action along with anxiolytic effects of its crude extract have already been justified. This investigation determined that bioactive compounds of the plant could be related to its actions and justify the ethnopharmacological importance of the plant. The results of the present investigation revealed the antinociceptive potential of the experimental PMFs of *N. plumbaginifolia*, where 3,3′,5,6,7,8-hexamethoxy-4′,5′-methylenedioxyflavone (**1**) 6,7,4′,5′-dimethylenedioxy-3,5,3′-trimethoxyflavone (**3**) and 3,3′,4′,5,5′,8-hexamethoxy-6,7-methylenedioxyflavone (**4**) produced significant, dose-dependent effect. The antinociceptive action of the test compounds involved opioid receptor, ATP-sensitive K^+^ channel as well as suppression of inflammatory mediators such as PGs, COX, LOX. Although the PMFs have been administered intragastrically and standard drugs intraperitoneally, the effect of compound **3** was greater than the respective standard drug in hot plate and acetic acid tests to some extent (% MPE and onset of writhing, respectively) for the positive response which could be considered a significant finding. Evaluation of neuropharmacological activities aided the elucidation of their anxiolytic activity. The study also demonstrated anxiolytic-like action involving benzodiazepine receptors without altering the locomotor responses at experimental doses. However, direct modulation of the receptors and nociceptive mediators by the experimental PMFs will be a greater part of interest. Considering the present findings into account, it may suggest that the PMFs of *N. plumabginifolia* could be suitable candidates for the development of analgesics as well as anxiolytic agents.

## Author contributions

The study was conceived and designed by BKD, MAR, and MSS. The experiments were carried out by MSS and SI. MSS and RBR performed data analysis. MSS, MAR, and RBR drafted the manuscript. SDS, LN, LCM, and MMRS have gone through the manuscript to edit and improved the quality of the manuscript significantly. The final content of the manuscript was revised and approved by all the authors.

### Conflict of interest statement

The authors declare that the research was conducted in the absence of any commercial or financial relationships that could be construed as a potential conflict of interest.

## Abbreviations

PMFs: Polymethoxyflavones
NSAIDs: non-steroidal anti-inflammatory drugs
ATP: Adenosine triphosphate
Na-CMC: sodium carboxymethyl cellulose
OECD: Organization for Economic Cooperation and Development
AVMA: American Veterinary Medical Association
icddr: b,International Center for Diarrhoeal Disease Research
b.w.: body weight
p.o.: per oral
i.p.: intraperitoneal
μL: microliter
mL: milliliter
cm: centimeter
mg: milligram
kg: kilogram
MA: mean ambulation
MPE: maximal possible analgesic effect
AUC: area under the curve
min: minimum
max: maximum
n: number of mice
NLX: naloxone
Gbc: glibenclamide
Flu: flumazenil
PG: prostaglandin
NO: nitric oxide
iNOS: Inducible nitric oxide synthase
CASP6: Caspase-6 precursor
cGMP: cyclic guanosine monophosphate
PG: prostaglandin
LOX: lipoxygenase
COX: cyclooxygenase
TNF-α: Tumor necrosis factor alpha
IL-8: Interleukin 8
IL-1β: Interleukin-1 beta
GABA: gamma aminobutyric acid
CNS: central nervous system.
