# Supplementary material for: Polymethoxyflavones from Nicotiana plumbaginifolia (Solanaceae) Exert Antinociceptive and Neuropharmacological Effects in Mice
Source: Front Pharmacol. 2018 Feb 20;9:85. doi: 10.3389/fphar.2018.00085 (PMC5826308; doi:10.3389/fphar.2018.00085)
Supplement: Supplementary file 1 [file DataSheet1.docx]

Supplementary Material

**Polymethoxyflavones from *Nicotiana plumbaginifolia* (Solanaceae) exert antinociceptive and neuropharmacological effects in mice**

**Md. Shafiullah Shajib^1^, Ridwan B. Rashid^2^, Long C. Ming^3,4^, Shanta Islam^1^,**

**Md. Moklesur R. Sarker^2^, Lutfun Nahar^5^, Satyajit D. Sarker^5^, Bidyut K. Datta^1^ and**

**Mohammad A. Rashid^6*^**

^1^ Department of Pharmacy, Stamford University Bangladesh, Dhaka, Bangladesh,

^2^ Department of Pharmacy, State University of Bangladesh, Dhaka, Bangladesh,

^3^ School of Pharmacy, KPJ Healthcare University College, Nilai, Malaysia,

^4^ Unit for Medication Outcomes Research and Education, Pharmacy, University of Tasmania, Hobart, TAS, Australia,

^5^ Faculty of Science, School of Pharmacy and Biomolecular Sciences, Liverpool John Moores University, Liverpool, United Kingdom,

^6^ Department of Pharmaceutical Chemistry, Faculty of Pharmacy, University of Dhaka, Dhaka, Bangladesh

*** Correspondence:**

Mohammad A. Rashid
rashidma@du.ac.bd

# Supplementary Tables

Supplementary Table 1 | Effect of diclofenac, compounds 1- 4 and pre-treatment of glibenclamide in acetic acid-induced writhing test

| **Treatment** | **Dose (mg/kg)** | **Onset time** | **Total number of writhing** | **% inhibition** |
| --- | --- | --- | --- | --- |
| Vehicle | - | 227.64 (206.24-244.65) | 116.00 (101.00-119.50) | - |
| Diclofenac | 10 | 236.95 (224.22-247.19) | 46.50^*^(35.00-58.00) | 59.77 |
| **1** | 12.5 | 229.35 (214.10-261.32) | 98.75 (83.00-115.50) | 15.81 |
| **1** | 25 | 281.62^*^ (268.20-296-93) | 79.20^*^ (66.50-84.50) | 31.68 |
| **2** | 12.5 | 228.11 (202.56-246.24) | 95.50^*^ (86.00-110) | 16.21 |
| **2** | 25 | 261.93^*^ (248.92-274.38) | 87.00^*^ (66.50-91.50) | 24.59 |
| **3** | 12.5 | 275.53^*^ (259.38-289.37) | 78.50^*^ (65.00-97.00) | 30.39 |
| **3** | 25 | 307.34^*^ (294.25-343.36) | 51.50^*^ (43.50-56.50) | 54.90 |
| **4** | 12.5 | 246.45 (223.50-256.37) | 97.00 (89.50-116.50) | 18.83 |
| **4** | 25 | 278.17^*^ (262.41-285.34) | 77.50^*^ (74.50-91.00) | 29.29 |
| Gbc | 10 | 218.95 (188.47-247.32) | 111.50 (102.50-115.00) | 3.67 |
| Gbc + Diclofenac | 10 + 10 | 220.04 (210.57-238.52) | 86.25^a^ (69.00-95.50) | 25.82 |
| Gbc + **1** | 10 + 25 | 235.80^b^ (221.40-248.67) | 103.00^b^ (91.00-116.50) | 9.96 |
| Gbc + **2** | 10 + 25 | 236.56^c^ (218.64-257.46) | 114.50^c^ (104.50-118.50) | 0.40 |
| Gbc + **3** | 10 + 25 | 256.46^d^ (231.73-285.04) | 83.50^d^ (75.50-91.00) | 28.02 |
| Gbc + **4** | 10 + 25 | 236.64^e^ (217.44-247-87) | 109.25^e^ (102.00-128.00) | 7.41 |

Values are presented as median (n = 6) with range (min-max). **1** = 3,3',5,6,7,8-hexamethoxy-4',5'-methylenedioxyflavone; **2** = exoticin; **3** = 6,7,4',5'-dimethylenedioxy-3,5,3'-trimethoxyflavone; **4** = 3,3',4',5,5',8-hexamethoxy-6,7-methylenedioxyflavone, Gbc = glibenclamide. ^*^ represents *p* < 0.01 compared to control group; ^a, b, c, d, e^ represents *p* < 0.01 compared to diclofenac (10 mg/kg), **1** (25 mg/kg), **2** (25 mg/kg), **3** (25 mg/kg) and **4** (25 mg/kg) respectively.

Supplementary Table 2 | Effect of diclofenac, compounds 1- 4 and pre-treatment of glibenclamide on writhing response at multiple time interval.

| **Treatment** | **Dose (mg/kg)** | **Number of writhing** | | | | | |
| --- | --- | --- | --- | --- | --- | --- | --- |
|  |  | **10 min** | **20 min** | **30 min** | **40 min** | **50 min** | **60 min** |
| Vehicle | - | 27.00  (25.00-33.00) | 24.00  (22.00-26.50) | 21.25  (15.50-28.50) | 17.00  (10.50-23.00) | 14.75  (11.00-19.00) | 9.50  (7.00-10.50) |
| Diclofenac | 10 | 12.00^*^  (10.50-13.50) | 12.75^*^  (7.50-14.00) | 7.25^*^  (4.00-12.00) | 5.00^*^  (3.00-9.50) | 3.75^*^  (2.00-8.50) | 3.75^*^  (2.50-8.00) |
| **1** | 12.5 | 22.50  (15.50-36.50) | 21.25  (18.50-27.50) | 19.25  (13.50-25.00) | 12.00  (10.50-17.00) | 9.50^*^  (8.00-14.00) | 9.50  (7.00-11.50) |
| **1** | 25 | 21.00^*^  (18.50-27.50) | 18.25  (14.00-24.00) | 12.50^*^  (9.50-16.50) | 8.00^*^  (7.50-10.50) | 6.75^*^  (5.00-8.50) | 7.25^*^  (5.50-8.50) |
| **2** | 12.5 | 22.50  (20.50-32.00) | 23.25  (19.00-26.00) | 17.50  (12.50-22.50) | 12.50  (11.50-15.00) | 11.25^*^  (6.50-11.50) | 9.25  (6.50-11.50) |
| **2** | 25 | 21.00^*^  (15.00-25.50) | 21.25  (15.50-26.50) | 15.75^*^  (12.50-17.50) | 9.50^*^  (7.50-11.50) | 9.00^*^  (4.00-12.00) | 6.75  (5.00-10.00) |
| **3** | 12.5 | 21.75^*^  (19.00-22.50) | 19.75  (14.00-31.00) | 13.50^*^  (11.50-15.50) | 9.25^*^  (5.00-13.00) | 8.00^*^  (6.00-9.50) | 8.75  (6.00-9.50) |
| **3** | 25 | 14.00^*^  (10.50-16.50) | 13.00^*^  (11.00-16.50) | 8.75^*^  (5.50-11.50) | 5.25^*^  (3.50-9.00) | 5.75^*^  (2.50-8.00) | 4.75^*^  (2.00-7.00) |
| **4** | 12.5 | 24.25  (23.00-29.00) | 23.25  (19.00-27.00) | 16.75  (13.00-26.00) | 12.00  (10.00-16.00) | 11.00^*^  (8.00-13.00) | 8.75  (8.00-10.50) |
| **4** | 25 | 20.75^*^  (16.00-27.50) | 20.50^*^  (15.00-23.50) | 12.25^*^  (10.00-14.50) | 10.50^*^  (4.00-11.00) | 8.50^*^  (7.50-12.00) | 8.00  (7.00-10.50) |
| Gbc | 10 | 27.25  (21.00-32.00) | 23.25  (18.50-25.00) | 22.00  (19.00-25.00) | 14.00  (13.00-15.00) | 14.25  (11.00-16.50) | 11.50  (10.50-13.00) |
| Gbc + Diclofenac | 10 + 10 | 19.50^a^  (18.00-25.00) | 18.00^a^  (14.00-23.00) | 14.25^a^  (11.00-23.00) | 12.75^a^  (7.50-15.50) | 10.00^a^  (5.50-13.00) | 7.25^a^  (6.00-12.00) |
| Gbc + **1** | 10 + 25 | 26.25  (20.50-31.00) | 22.00  (18.50-25.00) | 18.00^b^  (13.50-19.50) | 13.00^b^  (11.00-15.50) | 12.75^b^  (11.00-16.00) | 12.00^b^  (9.00-12.50) |
| Gbc + **2** | 10 + 25 | 25.50 ^c^  (23.00-30.00) | 22.75  (19.50-28.00) | 19.75^c^  (17.00-22.50) | 16.25^c^  (13.50-17.50) | 15.25^c^  (12.50-17.00) | 13.25  (10.00-17.00) |
| Gbc + **3** | 10 + 25 | 19.25^d^  (17.50-23.00) | 17.00 ^d^  (14.00-21.00) | 13.75^d^  (12.00-15.50) | 12.50^d^  (9.50-15.00) | 11.50^d^  (8.50-14.00) | 9.00^d^  (7.50-14.00) |
| Gbc + **4** | 10 + 25 | 26.50 ^e^  (24.50-28.00) | 22.25  (19.00-29.50) | 17.50^e^  (15.00-20.50) | 14.25^e^  (13.50-21.00) | 14.25^e^  (10.50-17.00) | 13.50^e^  (13.00-15.00) |

Values are presented as median (n = 6) with range (min-max). **1** = 3,3',5,6,7,8-hexamethoxy-4',5'-methylenedioxyflavone; **2** = exoticin; **3** = 6,7,4',5'-dimethylenedioxy-3,5,3'-trimethoxyflavone; **4** = 3,3',4',5,5',8-hexamethoxy-6,7-methylenedioxyflavone, Gbc = glibenclamide. ^*^represents *p* < 0.05, compared to control group. ^a, b, c, d, e^ represents *p* < 0.05, compared to diclofenac (10 mg/kg), **1** (25 mg/kg), **2** (25 mg/kg), **3** (25 mg/kg) and **4** (25 mg/kg) group, respectively.

| **Treatment** | **Dose (mg/kg)** | **Latency time (s)** | | | | | |
| --- | --- | --- | --- | --- | --- | --- | --- |
|  |  | **Pre-treatment** | **30 min** | **45 min** | **60 min** | **90 min** | **120 min** |
| Vehicle | - | 5.52  (4.98-6.63) | 6.01  (5.84-6.48) | 5.83  (5.41-6.38) | 5.97  (5.66-6.05) | 6.00  (5.76-6.84) | 6.04  (4.89-6.90) |
| Morphine | 5 | 5.92  (5.36-6.65) | 9.79^**^  (8.87-11.34) | 11.63^**^  (8.91-73.71) | 14.18^**^  (12.14-16.47) | 12.57^**^  (8.20-15.03) | 10.63^**^  (8.34-11.74) |
| **1** | 12.5 | 5.83  (5.33-6.74) | 6.75  (5.80-7.11) | 7.26^**^  (6.85-9.03) | 9.11^**^  (7.67-13.16) | 10.25^**^  (9.11-12.30) | 10.25^**^  (9.44-12.30) |
| **1** | 25 | 5.83  (5.15-6.42) | 7.17^**^  (6.36-7.74) | 7.73^**^  (6.92-8.80) | 9.96^**^  (7.47-13.14) | 12.84^**^  (10.04-14.00) | 12.62^**^  (10.80-13.63) |
| **2** | 12.5 | 6.17  (5.52-6.80) | 6.91^**^  (6.15-7.09) | 7.08^**^  (6.82-7.89) | 7.43^**^  (7.09-8.97) | 8.80^**^  (7.14-9.45) | 7.37^**^  (6.26-8.25) |
| **2** | 25 | 6.01  (5.38-6.37) | 6.64^*^  (6.00-6.87) | 6.91^**^  (6.45-7.91) | 8.79^**^  (7.34-9.60) | 10.48^**^  (9.81-11.61) | 9.90^**^  (8.75-11.33) |
| **3** | 12.5 | 6.91  (6.65-7.80) | 7.10^**^  (6.82-8.15) | 11.09^**^  (8.50-12.60) | 11.09^**^  (8.50-12.60) | 11.30^**^  (8.33-12.70) | 11.85^**^  (10.46-12.89) |
| **3** | 25 | 5.89  (5.47-6.30) | 6.78^**^  (5.87-7.05) | 8.27^**^  (7.29-9.65) | 13.48^**^  (12.11-14.08) | 12.90^**^  (11.88-13.48) | 13.39^**^  (10.32-14.05) |
| **4** | 12.5 | 6.02  (5.09-6.55) | 6.84  (5.80-7.27) | 7.26^**^  (6.20-8.52) | 9.38^**^  (7.33-10.48) | 9.64^**^  (8.77-11.46) | 10.83^**^  (10.36-11.35) |
| **4** | 25 | 5.86  (5.19-6.47) | 6.71  (6.10-7.59) | 7.80^**^  (6.99-9.90) | 10.33^**^  (9.11-11.56) | 11.87^**^  (9.00-13.09) | 11.99^**^  (9.51-13.21) |
| NLX | 2 | 5.77  (5.03-6.96) | 6.38  (5.31-6.91) | 6.48  (5.06-6.94) | 6.12  (5.06-6.94) | 5.86  (5.12-6.96) | 6.10  (5.15-8.56) |
| NLX + Morphine | 2 + 5 | 6.04  (5.36-6.35) | 6.14^a^  (5.73-6.77) | 6.97^a^  (5.70-7.52) | 8.31^a^  (6.19-9.52) | 7.47^a^  (6.04-8.30) | 7.07^a^  (6.14-7.72) |
| NLX + **1** | 2 + 25 | 6.16  (5.16-6.93) | 6.77  (5.25-7.10) | 6.86  (6.14-8.82) | 7.24^b^  (5.74-9.20) | 8.20^b^  (5.98-10.46) | 8.27^b^  (7.12-10.61) |
| NLX + **2** | 2 + 25 | 5.77  (5.06-6.23) | 6.29  (5.86-6.87) | 6.96  (5.96-7.23) | 7.35^c^  (6.56-7.92) | 8.63^c^  (6.99-9.78) | 8.54^c^  (7.11-9.34) |
| NLX + **3** | 2 + 25 | 5.97  (5.46-6.57) | 6.57  (6.03-6.98) | 6.93^d^  (6.01-7.75) | 8.68^d^  (7.71-10.14) | 9.01^d^  (8.05-11.57) | 9.74^d^  (8.46-10.71) |
| NLX + **4** | 2 + 25 | 6.00  (5.43-6.37) | 6.34  (5.94-6.71) | 7.22  (6.26-8.03) | 8.75^e^  (7.98-9.57) | 9.71  (8.29-10.74) | 9.73^e^  (8.19-11.28) |

Supplementary Table 3 | Effect of compounds 1- 4, morphine and pre-treatment of naloxone on latency time in hot plate test

Values are presented as median (n = 6) with range (min-max). **1** = 3,3',5,6,7,8-hexamethoxy-4',5'-methylenedioxyflavone; **2** = exoticin; **3** = 6,7,4',5'-dimethylenedioxy-3,5,3'-trimethoxyflavone; **4** = 3,3',4',5,5',8-hexamethoxy-6,7-methylenedioxyflavone, NLX = naloxone. ^*^, ^**^ represents *p* < 0.05 and *p* < 0.01, compared to control group, respectively. ^a, b, c, d, e^ represents *p* < 0.05, compared to morphine (5 mg/kg), **1** (25 mg/kg), **2** (25 mg/kg), **3** (25 mg/kg) and **4** (25 mg/kg), respectively.

Supplementary Table 4 | Effect of compounds 1- 4, morphine and pre-treatment of naloxone on latency time in tail immersion test

| **Treatment** | **Dose (mg/kg)** | **Latency time (s)** | | | | | |
| --- | --- | --- | --- | --- | --- | --- | --- |
|  |  | **Pre-treatment** | **30 min** | **45 min** | **60 min** | **90 min** | **120 min** |
| Vehicle | - | 2.44  (2.05-3.18) | 2.88  (2.27-3.52) | 3.11  (2.10-3.70) | 2.98  (2.37-3.45) | 2.84  (2.52-3.58) | 2.78^*^  (2.32-3.86) |
| Morphine | 5 | 2.41  (2.03-3.06) | 7.91^*^  (7.00-9.04) | 9.85^*^  (9.11-11.06) | 10.69^*^  (10.01-12.36) | 5.11^*^  (4.01-6.03) | 5.01 ± 0.34 |
| **1** | 12.5 | 2.32  (2.21-2.49) | 2.61  (2.45-2.89) | 2.90  (2.74-3.26) | 3.13  (2.97-3.36) | 3.23  (3.10-3.63) | 3.36  (3.22-3.64) |
| **1** | 25 | 2.67  (2.00-3.17) | 3.02  (2.46-3.42) | 3.22  (2.73-3.79) | 3.45  (3.04-3.91) | 3.59  (3.14-4.03) | 3.77  (3.17-4.14) |
| **2** | 12.5 | 2.87  (2.36-3.29) | 3.39  (2.52-3.98) | 3.42  (2.82-3.76) | 3.49  (2.94-4.15) | 3.62  (3.09-4.41) | 3.53  (2.51-4.68) |
| **2** | 25 | 2.71  (2.26-3.10) | 3.17  (2.53-3.67) | 3.45  (2.16-4.29) | 3.55  (3.08-4.03) | 3.75  (3.35-4.17) | 3.73  (3.16-4.14) |
| **3** | 12.5 | 2.93  (2.70-3.17) | 3.41  (2.99-3.61) | 3.84^*^  (3.59-3.92) | 4.51^*^  (4.16-5.05) | 5.09^*^  (4.85-5.61) | 5.53^*^  (5.02-6.20) |
| **3** | 25 | 2.37  (2.16-3.04) | 3.14  (2.46-3.77) | 4.45^*^  (3.87-4.89) | 5.06^*^  (4.76-5.37) | 5.53^*^  (5.36-6.11) | 5.97^*^  (5.55-6.70) |
| **4** | 12.5 | 2.30  (2.10-2.52) | 2.64  (2.32-2.98) | 2.68  (2.27-3.07) | 2.64  (2.55-3.17) | 2.91  (2.71-3.55) | 3.12  (2.86-3.85) |
| **4** | 25 | 2.39  (2.09-2.84) | 2.89  (2.28-3.35) | 3.17  (2.66-3.58) | 3.31  (2.41-3.89) | 3.31  (2.85-3.61) | 3.48  (2.86-3.94) |
| NLX | 2 | 2.35  (2.06-3.37) | 2.78  (2.03-3.70) | 3.27  (2.03-3.43) | 3.09  (2.15-3.59) | 3.03  (2.43-3.78) | 3.08  (2.02-3.47) |
| NLX + Morphine | 2 + 5 | 2.22  (2.01-3.02) | 2.76^a^  (2.24-3.04) | 3.65^a^  (3.16-4.19) | 3.71^a^  (3.03-4.62) | 2.90^a^  (2.24-3.60) | 2.50^a^  (2.15-3.90) |
| NLX + **3** | 2 + 25 | 2.51  (2.31-2.81) | 2.86  (2.57-3.24) | 2.82^b^  (2.53-3.70) | 2.80^b^  (2.37-3.90) | 3.17^b^  (2.86-3.64) | 3.40^b^  (2.34-3.69) |

Values are presented as median (n = 6) with range (min-max). **1** = 3,3',5,6,7,8-hexamethoxy-4',5'-methylenedioxyflavone; **2** = exoticin; **3** = 6,7,4',5'-dimethylenedioxy-3,5,3'-trimethoxyflavone; **4** = 3,3',4',5,5',8-hexamethoxy-6,7-methylenedioxyflavone, NLX = naloxone. ^*^ represents *p* < 0.01, compared to control group. ^a, b^ represents *p* < 0.01, compared to morphine (5 mg/kg) and **3** (25 mg/kg), respectively.

# Supplementary Figure





**Supplementary Figure 1.** **Effects of compounds 1-4 and morphine on formalin-induced nociception at multiple time interval**. The panels show time versus responses curve of vehicle, morphine and compound **1** (**A**), **2** (B), **3** (**C**) or **4** (**D**) treated groups. **1** = 3,3',5,6,7,8-hexamethoxy-4',5'-methylenedioxyflavone; **2** = exoticin; **3** = 6,7,4',5'-dimethylenedioxy-3,5,3'-trimethoxyflavone; **4** = 3,3',4',5,5',8-hexamethoxy-6,7-methylenedioxyflavone. Values are presented as median (n = 6) with range (min-max). ^*^ represents *p* < 0.05 compared to control group at the same time interval.
